# Thermally Activated and Aggregation‐Regulated Excitonic Coupling Enable Emissive High‐Lying Triplet Excitons**

**DOI:** 10.1002/anie.202206681

**Published:** 2022-07-04

**Authors:** Tao Wang, Joydip De, Sen Wu, Abhishek Kumar Gupta, Eli Zysman‐Colman

**Affiliations:** ^1^ Organic Semiconductor Centre EaStCHEM School of Chemistry University of St Andrews St Andrews KY16 9ST UK

**Keywords:** Excitonic Coupling, Higher-Lying Triplet Excitons, Host–Guest Systems, Room-Temperature Phosphorescence

## Abstract

Room‐temperature phosphorescence (RTP) originating from higher‐lying triplet excitons remains a rather rarely documented occurrence for purely organic molecular systems. Here, we report two naphthalene‐based RTP luminophores whose phosphorescence emission is enabled by radiative decay of high‐lying triplet excitons. In contrast, upon cooling the dominant phosphorescence originates from the lowest‐lying triplet excited state, which is manifested by a red‐shifted emission. Photophysical and theoretical studies reveal that the unusual RTP results from thermally activated excitonic coupling between different conformations of the compounds. Aggregation‐regulated excitonic coupling is observed when increasing the doping concentration of the emitters in poly(methylmethacrylate) (PMMA). Further, the RTP quantum efficiency improves more than 80‐fold in 1,3‐bis(*N*‐carbazolyl)benzene (mCP) compared to that in PMMA. This design principle offers important insight into triplet excited state dynamics and has been exploited in afterglow‐indicating temperature sensing.

## Graphical abstract

Two naphthalene‐based luminophores that exhibit room‐temperature phosphorescence (RTP) from a higher‐lying triplet excited state are reported. Experimental and theoretical investigations reveal that unusual RTP originates from the multiple accessible triplet excited‐stated conformers, regulated by thermal activation and aggregation. This unique RTP family enables functional temperature sensing.

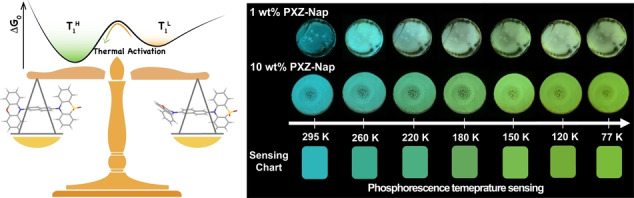

## Introduction

Organic room‐temperature phosphorescence (RTP) is the radiative emission of triplet excitons,^[1]^ which occurs with a phosphorescence lifetime, *τ*_ph_, ranging from microseconds to milliseconds. In the past decade, the development and study of organic RTP materials have gained increasing attention due to their potential in applications across diverse fields such as optoelectronics,^[2]^ lasers,^[3]^ sensing,^[4]^ imaging,^[5]^ data storage,^[6]^ and X‐ray scintillators.^[7]^ To date, a plethora of design motifs have been proposed, mainly based on the modulation of intramolecular and/or intermolecular interactions.^[8]^ These RTP design principles are primarily based on improving the intersystem crossing (ISC) efficiency and/or suppressing the non‐radiative and oxygen quenching deactivation of the triplet excitons. For example, ISC efficiency has been shown to be enhanced via the incorporation of heavy atoms,^[9]^ the breaking of conjugation,[^2a^, ^4b^, ^10^] and the introduction of a charge‐transfer (CT) bridge motif[^5c^, ^11^] and resonance linkage^[12]^ (intramolecular modulation). Also, matrix rigidification,^[13]^ molecular aggregation,^[14]^ and polymerization^[15]^ have each been demonstrated to suppress the non‐radiative decay of triplet excitons (intermolecular interactions) for achieving efficient RTP systems.

**Figure 1:**
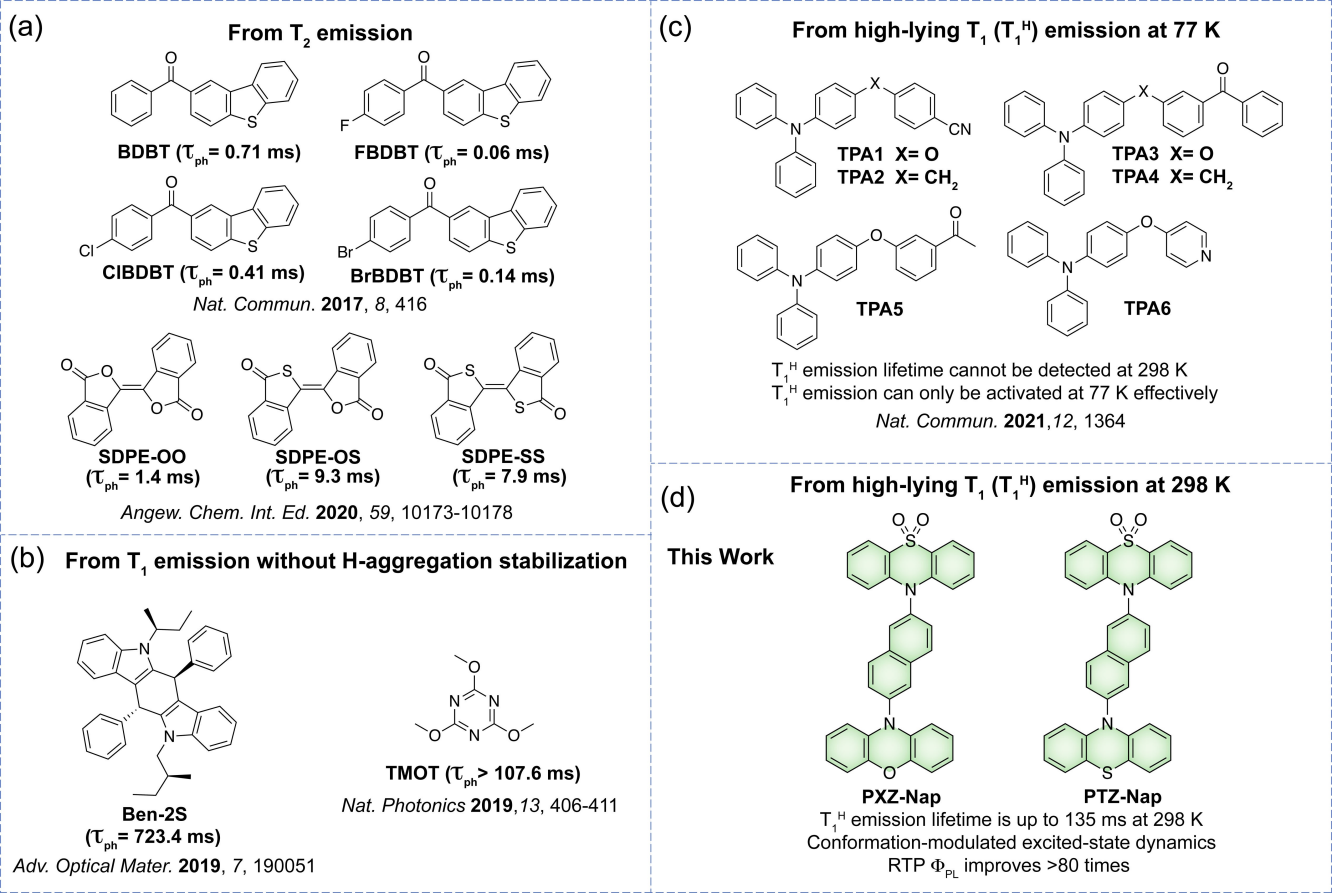
Chemical structures of reported anomalous RTP phosphors (a)–(c): a) Emissive T_2_ in crystals. b) Emissive T_1_ in crystals, whose energy is higher than H‐aggregation stabilized triplet state (T_1_*). c) Emissive T_1_^H^ at 77 K from decoupled donor–acceptor (D–A) motifs in PMMA. d) This work: emissive T_1_^H^ at 298 K from conjugated D–A compounds investigated with multiple accessible conformations. Lifetimes in parentheses represent the excitonic decay from higher‐lying triplet states at room temperature.

Consequently, most reported RTP research has focused on enhancing RTP efficiency (i.e., photoluminescence quantum yield, Φ_PL_) and *τ*_ph_. To date, there are only a few documented examples where RTP originates from a higher‐lying triplet excited state.[^10^, ^16^] For instance, Tang and co‐workers reported several benzothiophene derivatives showing dual phosphorescence, which was assumed to originate from both the first (T_1_) and second (T_2_) triplet excited states (Figure 1a).^[16b]^ Huang and co‐workers designed materials based on carbazole that enabled dual phosphorescence from T_1_ and an *H*‐aggregation stabilized species (T_1_*, T_1_*<T_1_) (Figure 1b).[^16c^, ^16d^] However, this dual phosphorescence was only observed to occur in crystals, making it difficult to identify the implication of any higher‐lying triplet states in the emission processes. Recently, Zhang and co‐workers developed a series of triphenylamine‐*sp* ^3^ linker‐acceptor motifs exhibiting phosphorescence that was attributed to a high‐lying T_1_ (T_1_^H^) state associated with the electronically decoupled donor and acceptor groups in the compound (Figure 1c).^[10]^ Nevertheless, emission from the T_1_^H^ state could only be observed at low temperature (<250 K). It thus remains a formidable challenge to design RTP materials whose emission originates from higher‐lying triplet excited states.

Emission from CT states in donor‐acceptor compounds is sensitive to the solvent polarity and originates from the solvent‐stabilized excited states of emissive conformers.^[17]^ We hypothesized that it may be possible to achieve RTP from higher‐lying triplet states by accessing non‐equilibrium conformers in the excited state in the solid state, where the interconversion barrier from one conformer to another must be overcome for excitonic coupling to occur. Based on this concept, we report two conjugated RTP molecules, PXZ‐Nap and PTZ‐Nap (Figure 1d) using phenoxazine (PXZ) and phenothiazine (PTZ) as donors, which have both been observed to show conformational dynamics under external stimuli,^[18]^ and a substituted naphthalene (Nap) as the acceptor. The decoration of a 10*H*‐phenothiazine‐5,5‐dioxide unit on the Nap distal to the donor is anticipated to modulate the excited state dynamics and to accelerate the ISC process owing to the presence of the sulfur atom.^[19]^ We found that when the two compounds are doped in poly(methylmethacrylate) (PMMA) or 1,3‐bis(*N*‐carbazolyl)benzene (mCP) (1 wt %), RTP from higher‐lying triplet states occurs due to a thermally activated excitonic coupling between different conformers. Moreover, the RTP quantum efficiency of the emitters doped in mCP (1 wt %) improved over 80‐fold compared to that in PMMA. The photophysics at a higher doping of 10 wt % in PMMA films revealed that there exist aggregation‐regulated intermolecular interactions that offer a route to modulating T_1_^H^ and low‐lying T_1_ (T_1_^L^). Finally, we have exploited the anomalous RTP behavior to produce a temperature sensor.

## Results and Discussion

The synthesis of PXZ‐Nap and PTZ‐Nap is outlined in Scheme S1. The molecular structures and purity of the two compounds were confirmed by a combination of ^1^H and ^13^C nuclear magnetic resonance spectroscopy (NMR), high‐resolution mass spectrometry (HRMS), elemental analysis (EA), melting point determination and high‐performance liquid chromatography (HPLC) (Figures S1–S13).

**Figure 2:**
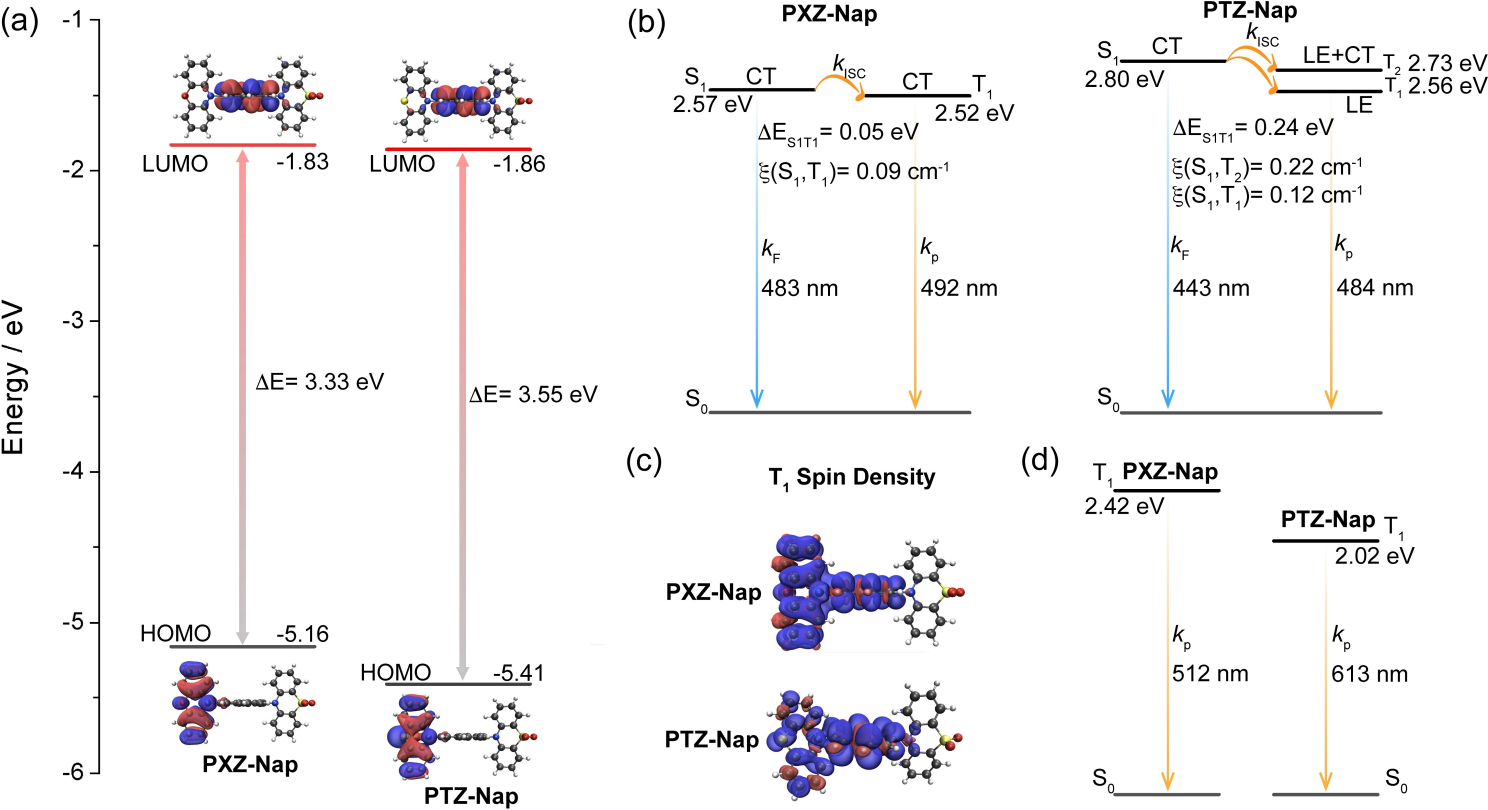
a) Frontier molecular orbitals (isovalue: 0.02) and b) vertical excitation energy levels of PXZ‐Nap and PTZ‐Nap calculated using the optimized S_0_ geometry in the gas phase at the PBE0/6‐31G(d,p) level. c) T_1_ Spin density distributions (isovalue: 0.0004) and d) T_1_ vertical emission energies of PXZ‐Nap and PTZ‐Nap calculated in the gas phase at the T_1_ optimized geometry at the uPBE0/6‐31G(d,p) level.

We first modelled the optoelectronic properties of PXZ‐Nap and PTZ‐Nap in the gas phase using density functional theory (DFT) with the PBE0 functional^[20]^ and the 6‐31G(d,p) basis set^[21]^ (Figure 2). PXZ‐Nap and PTZ‐Nap possess similar lowest unoccupied molecular orbitals (LUMOs) that are localized on the Nap and highest occupied molecular orbitals (HOMOs) that are localized on the donor PXZ or PTZ, respectively (Figure 2a). The destabilized HOMO of PXZ‐Nap indicates that in these compounds PXZ is the stronger donor group. The dihedral angles of PXZ and PTZ with Nap in PXZ‐Nap and PTZ‐Nap at the optimized ground state (S_0_) geometry are 79.92° and 99.27° (Figure S14), respectively, revealing compounds with weakly electronically coupled donors to the Nap. Time‐dependent DFT (TD‐DFT) calculations at the same level of theory reveal that the lowest singlet excited state (S_1_) of each compound has a charge‐transfer (CT) character (Figures 2b and S15). Although PXZ‐Nap and PTZ‐Nap possess similar T_1_ energies, the nature of the T_1_ states in these compounds is distinct, being CT for PXZ‐Nap and locally excited (LE) for PTZ‐Nap, which are highly dependent on a specific excited‐state structure. However, triplet spin density distributions at the optimized T_1_ geometry indicate the T_1_ of both possess LE character (Figure 2c). At the relaxed S_1_ geometry there is a larger S_1_–T_1_ spin‐orbit coupling (SOC) matrix element (0.12 cm^−1^) in PTZ‐Nap due to the presence of the additional heavy sulfur atom than in PXZ‐Nap (0.09 cm^−1^). The predicted phosphorescence of PXZ‐Nap and PTZ‐Nap at their respective relaxed T_1_ geometries decrease to 2.42 eV and 2.02 eV, respectively, compared with the excitation T_1_ energies obtained from the TD‐DFT calculations at the S_0_ geometry (Figure 2d).

Next, the energies of the frontier molecular orbitals were inferred from the electrochemical behavior of PXZ‐Nap and PTZ‐Nap, measured using cyclic voltammetry (CV) and differential pulse voltammetry (DPV), in deaerated DMF with 0.1 M tetra‐*n*‐butylammonium hexafluorophosphate as the supporting electrolyte. The oxidation/reduction potentials (*E* ^ox^/*E* ^red^) of PXZ‐Nap and PTZ‐Nap determined from the DPV peaks are 0.79 eV/−2.09 eV and 0.78 eV/−2.09 eV vs. SCE,^[22]^ respectively (Figure S16a). Thus, PXZ‐Nap and PTZ‐Nap show similar HOMO values of −5.14 eV and −5.13 eV, respectively, despite the different donors,^[23]^ and the same LUMO value of −2.26 eV (Table S1), the result of the LUMO being localized on the Nap core.^[24]^ The results at first did not seem to align with the gas phase calculations (Figure 2a). However, a closer inspection of the DFT calculations of PTZ‐Nap, now employing a DMF continuum model, indicates that a quasi‐axial conformer is responsible for the destabilized HOMO (Figure S17); indeed, accessible quasi‐equatorial and axial conformations of PTZ have been previously reported.^[25]^ Okazaki et al. have also reported a series of PTZ‐containing molecules that exists in different conformations, with corresponding HOMO values that vary from −5.59 eV to −5.20 eV.^[23b]^

The UV/Vis absorption spectra show a weak CT transition at around 400 nm for both compounds (Figure S16b). The optical gaps (*E*_g_) of PXZ‐Nap and PTZ‐Nap estimated from the onsets of absorption spectra in toluene are 2.92 eV and 3.10 eV, respectively, which are almost same values as those measured in DMF (≈3.1 eV). We hypothesized that variation in *E*_g_ is caused by the existence of different conformers. The CT character of the emissive excited state was evidenced from the positive solvatochromism of the photoluminescence (PL) spectra (Figures S16c and S16d) and the accompanied increased PL lifetimes (Figure S18, Table S2).

**Figure 3:**
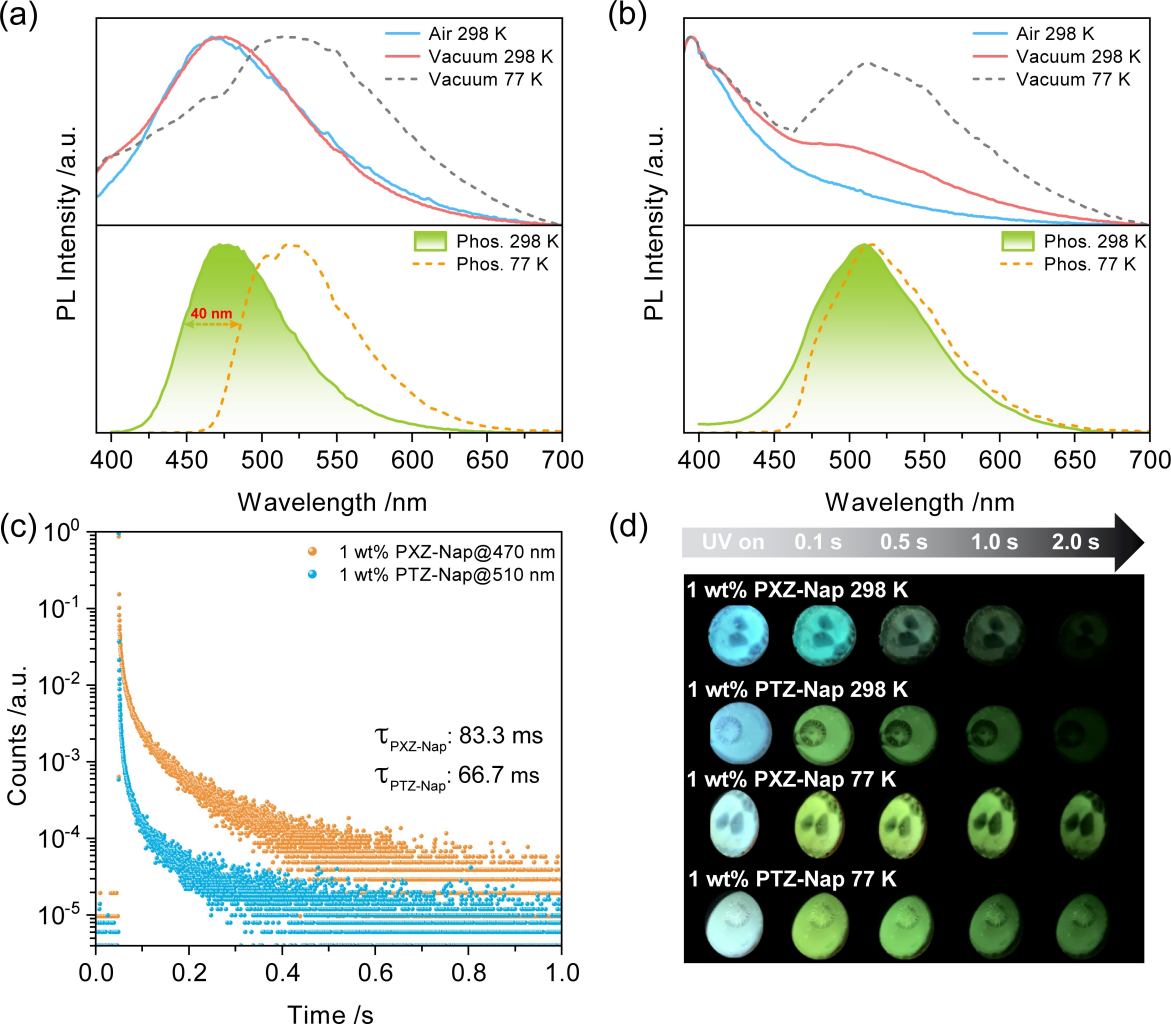
PL spectra of a) 1 wt % PXZ‐Nap and b) 1 wt % PTZ‐Nap in PMMA at 298 K and 77 K; top: steady‐state PL spectra (*λ*_exc_=370 nm); bottom: phosphorescence spectra collected using multichannel scaling (MCS) mode (*λ*_exc_: 379 nm; time‐gated window: 10–200 ms for 298 K; 0.1–1.5 s for 77 K). c) RTP lifetime decay profiles of 1 wt % PXZ‐Nap and 1 wt % PTZ‐Nap in PMMA. d) Images showing phosphorescence afterglows at 298 K and 77 K under vacuum (excitation source: 365 nm UV torch).

**Figure 4:**
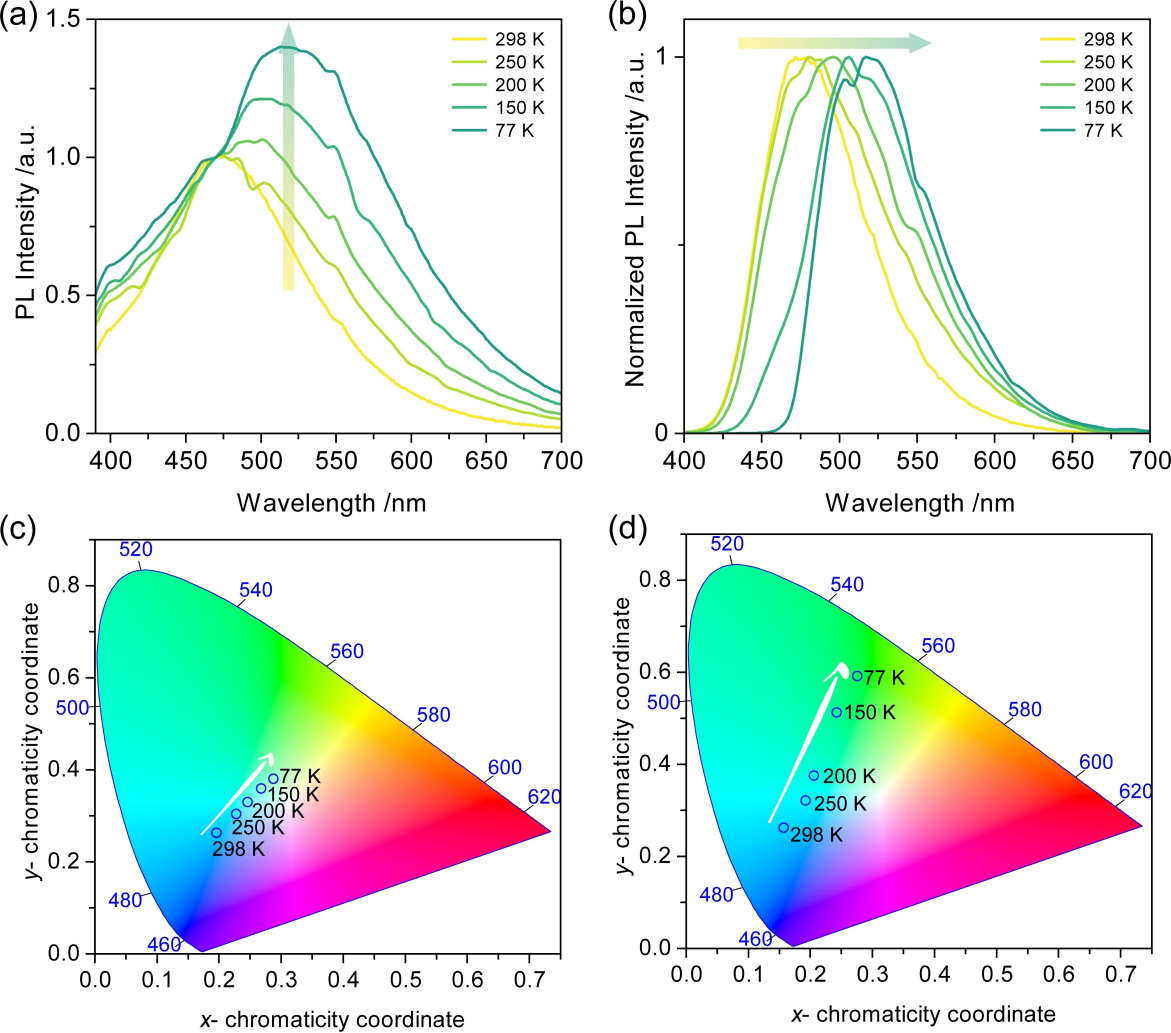
Temperature‐dependent a) steady‐state PL and b) normalized phosphorescence spectra of 1 wt % PXZ‐Nap in PMMA. Time‐gated window: 10–200 ms for 298 K; 0.1–1 s for 250 K, 200 K and 150 K; 0.1–1.5 s for 77 K). Time‐dependent CIE coordinates of c) steady‐state PL and d) phosphorescence emissions of 1 wt % PXZ‐Nap in PMMA.

We next investigated the photophysical properties of PXZ‐Nap and PTZ‐Nap in PMMA at 1 wt % doping. At this low doping concentration intermolecular interactions will only negligibly affect triplet excited dynamics while the impregnation of the emitters in a solid‐state matrix will serve to suppress non‐radiative decay. The steady‐state PL spectra of PXZ‐Nap in air and under vacuum (at 298 K) are broad, unstructured, and centered at 470 nm, which are characteristic of emission from a CT state (Figure 3a). At 77 K, there is the emergence of a second emission band at ≈510 nm that is assigned to phosphorescence. The PL lifetime of PXZ‐Nap under vacuum is 7.55 ns (Figure S19, Table S3). Time‐gated PL measurements (10 ms delay) detected the RTP spectrum of PXZ‐Nap (Figure 3a), which is centered also around 470 nm but is narrower and its decay has an associated *τ*_ph_ of 83.3 ms (Figure 3c). Such a long lifetime rules out thermally activated delay fluorescence (TADF) as the origin of this delayed emission. The low‐temperature phosphorescence (LTP) emission at 77 K is centered at 520 nm and possesses a slightly structured character, implying emission from a state with dominant LE character, which coincides with the calculations (Figure 2c). The difference in energy (0.27 eV) of the RTP and LTP, estimated from the onsets of phosphorescence emission spectra, suggests that they originate from two distinct conformers. Indeed, the blue RTP (T_1_^H^) and green LTP (T_1_^L^) afterglows provide stark evidence of emission from two different T_1_ states (Figure 3d). Temperature‐dependent steady‐state PL and phosphorescence studies show the gradually red‐shifted and intensified emission band centered at 525 nm (Figure 4a) and red‐shifted phosphorescence emission (Figure 4b), respectively, further demonstrating that temperature is the key to excitonic communication between T_1_^H^ and T_1_^L^. The related Commission Internationale de l′Éclairage (CIE) diagrams also reflect the gradual emission color change with changing temperature (Figures 4c and d). Temperature‐dependent lifetime studies (Figures S20a and S20b) reveal that the delayed emission is not thermally activated, providing further evidence that the origin of the long‐lived luminescence at 470 nm results from RTP and not from TADF; in fact, only at 77 K is there a slight decrease in the excited state lifetime (Figure S20b), which is attributed to the significantly suppressed T_1_^H^ emission (Figure 4b). Phosphorescence emission at 200 K measured at different time‐gated windows indicates variable excited‐state dynamics (Figure S21), also implying the existence of T_1_^H^ and T_1_^L^.

The picture for PTZ‐Nap is different. The steady‐state PL under air is blue‐shifted and structured, centered at 400 nm, characteristic of emission from an LE state; notably, there is also a broad emission tail (Figure 3b). The emission lifetime is 5.19 ns (Figure S19b). When placed under vacuum at 298 K, the structured emission beyond 475 nm is strengthened, which is strongly enhanced at 77 K. RTP emission can be observed in the range of 450–650 nm (Figure 3b), with an associated *τ*_ph_ of 66.7 ms. The LTP at 77 K has a similar emission but narrower profile compared to the RTP; however, the *τ*_ph_ (628 ms) is much longer (Figure S19c and Table S3). Similar RTP and LTP afterglows of 1 wt % PTZ‐Nap in PMMA indicate essentially two degenerate triplet states (Figure 3d). Temperature‐dependent photophysical investigations also reveal essentially indistinguishable T_1_^H^ and T_1_^L^ (Figure S22).

**Figure 5:**
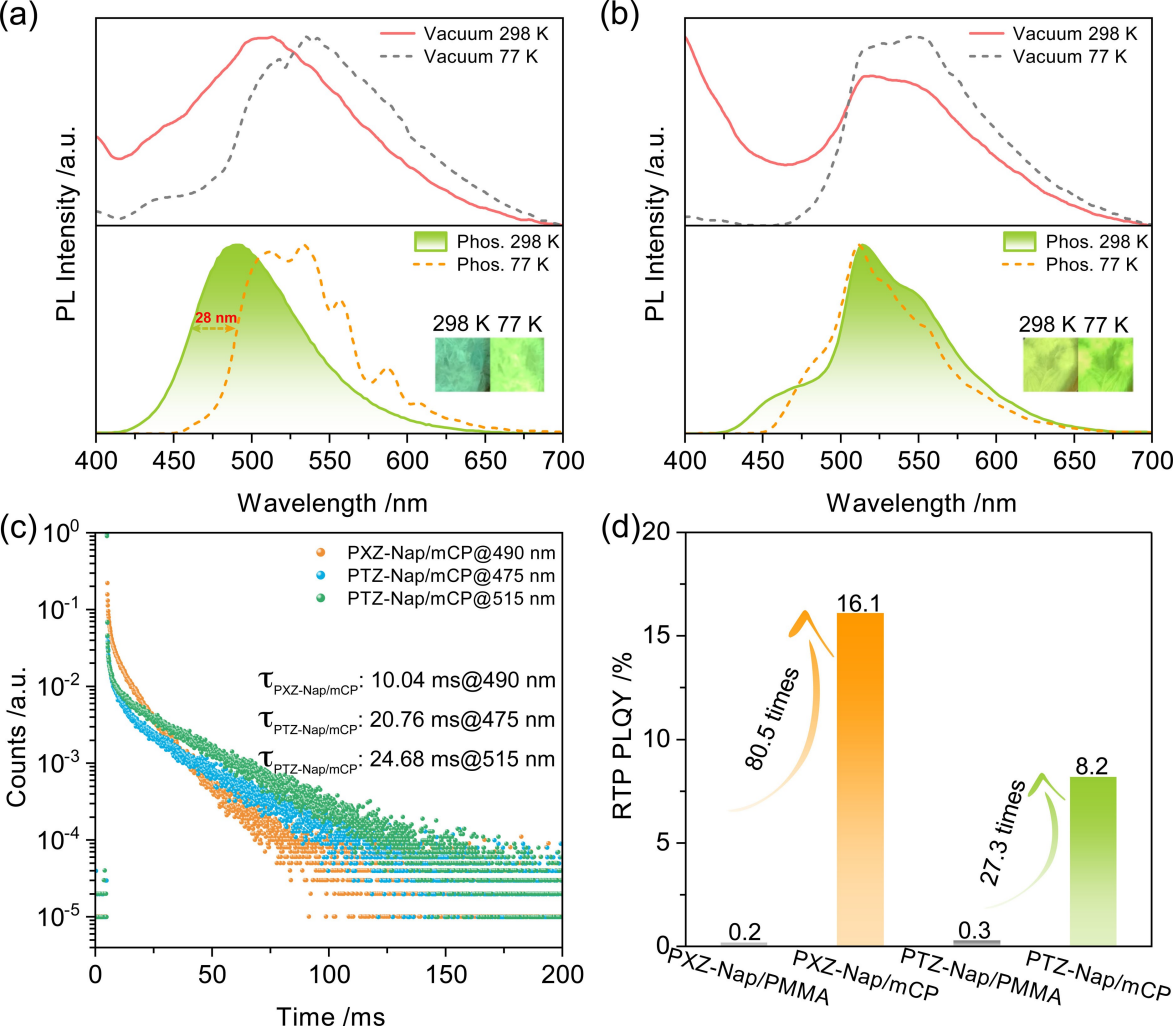
PL spectra of a) PXZ‐Nap/mCP and b) PTZ‐Nap/mCP at 298 K and 77 K; top: steady‐state emission spectra (*λ*_exc_=370 nm); bottom: phosphorescence spectra collected using MCS mode (*λ*_exc_=379 nm; time‐gated window: 10–200 ms for 298 K; 0.1–2.0 s for 77 K); inset: photos of phosphorescence afterglows excited by 365 nm UV torch. c) RTP lifetime decay profiles of PXZ‐Nap/mCP and PTZ‐Nap/mCP. d) RTP Φ_PL_ values of 1 wt % PXZ‐Nap and 1 wt % PTZ‐Nap in PMMA and mCP.

We then investigated the photophysics of PXZ‐Nap and PTZ‐Nap as 1 wt % doped films in mCP, a suitably high triplet energy host matrix (T_1_=3.0 eV),^[26]^ such that excitons would be confined onto the RTP materials. The photophysical data acquired under vacuum of PXZ‐Nap and PTZ‐Nap are summarized in Table S4. The steady‐state PL behavior of PXZ‐Nap and PTZ‐Nap in mCP is similar to that observed in PMMA (Figure 5). Notably, the low‐temperature PL is red‐shifted compared to the room temperature PL, which we contend is due to the dominant T_1_^L^ emission at 77 K as conformational dynamics are expected to be essentially arrested at this temperature. The RTP spectrum of PXZ‐Nap, collected with a time‐gated window of 10–100 ms, is red‐shifted (*λ*_em_=490 nm) and the *τ*_ph_ is shortened to 10.04 ms compared to that in 1 wt % doped PMMA (Figure 5c). A much more structured LTP emission at 77 K is observed (Figure 5a) compared to that of PXZ‐Nap in PMMA (Figure 3a). The enhanced LE character (≈400 nm) results from host‐guest interactions between PXZ‐Nap and mCP.

For PTZ‐Nap, a new high‐energy RTP emission band at around 425–500 nm can be observed, with an associated *τ*_ph_ of 20.76 ms (Figure 5c). At 77 K, this emissive triplet state is suppressed compared to the main emission band; however, the lifetime at 475 nm significantly increases to 1.5 s (Figure S23). The Φ_PL_ values of PXZ‐Nap and PTZ‐Nap are improved to 10.8 % and 7.7 % in air compared to those in 1 wt % doped PMMA (1.3 % and 5.3 %, Tables S3), respectively, values that are enhanced to 26.9 % and 15.9 % under vacuum, respectively (Table S4). Therefore, the Φ_PL_ associated with just the RTP (Φ_RTP_) of PXZ‐Nap and PTZ‐Nap in mCP are no smaller than 16.1 % and 8.2 %, respectively. Compared with the Φ_PL_ values of PXZ‐Nap and PTZ‐Nap in doped PMMA films, those in doped mCP films are enhanced 80.5 and 27.3 times, respectively (Figure 5d). Considering that the triplet energies of PXZ‐Nap and PTZ‐Nap (Table S4) are lower than that of mCP (T_1_=3.0 eV), the RTP efficiency enhancement benefits from the Dexter energy transfer process occurring between the T_1_ states of mCP and the guest emitters.^[27]^ These results demonstrate that different host‐guest interactions can regulate triplet excited dynamics.

**Figure 6:**
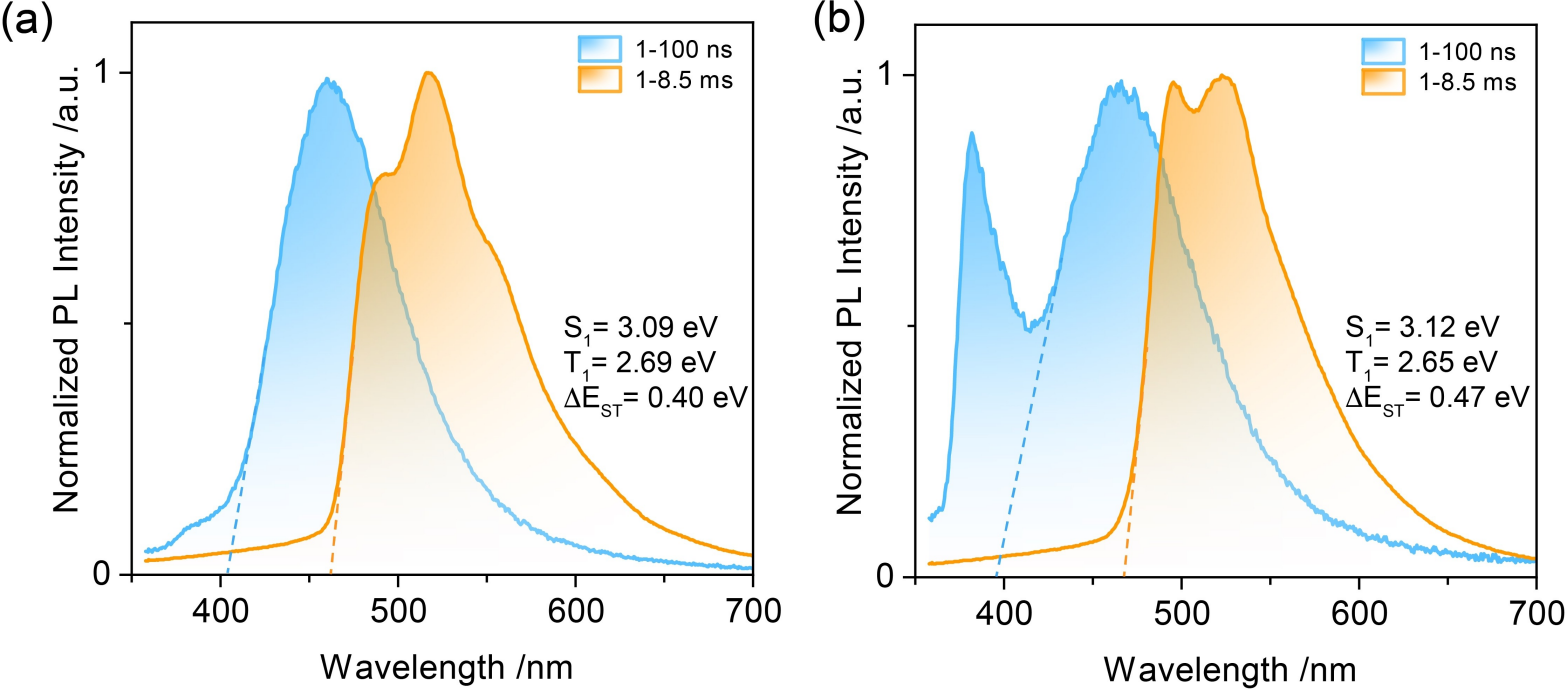
Prompt (1–100 ns) and delayed (1–8.5 ms) emission spectra of a) PXZ‐Nap and b) PTZ‐Nap in 2‐MeTHF at 77 K (*λ*_exc_=343 nm); concentration: 1×10^−6^ M.

To exclude that the triplet emission at around 525 nm originates from an aggregate, the prompt and delayed emission spectra were investigated in dilute 2‐MeTHF glass (1×10^−6^ M) at 77 K (Figure 6). Notably, the structured phosphorescence in 2‐MeTHF at 77 K for both compounds are at about the same energy, and also of similar energy to those measured in PMMA and mCP, which demonstrates that the phosphorescence from T_1_^L^ does not originate from an aggregate. The Δ*E*_ST_ values of PXZ‐Nap and PTZ‐Nap between S_1_ and T_1_^L^ in 2‐MeTHF glass are 0.40 eV and 0.47 eV, respectively. Despite the difference between the measured Δ*E*_ST_ in 2‐MeTHF compared with the predicted value (Figure 2b), we can clearly see that in doped PMMA and mCP films Δ*E*_ST_ between S_1_ and T_1_^H^ is almost degenerate, it is these values that coincide with the theoretical predictions. Notably, there is significant LE emission from **PTZ** at around 380 nm^[28]^ for PTZ‐Nap (Figure 6b) revealing that electron transfer to the CT state is slow compared to radiative decay. The experimentally calculated Δ*E*_ST_ at 77 K deviates from the computed values (0.05 eV and 0.24 eV, respectively, Figure 2b), which we attribute to different accessible relaxed conformers at room temperature compared to those at 77 K. Combining the above‐discussed results in PMMA, mCP and 2‐MeTHF glass, we rationally conclude that the T_1_ emission can be regulated by the host‐guest interactions that govern the conformational landscape of PTZ‐Nap.

**Figure 7:**
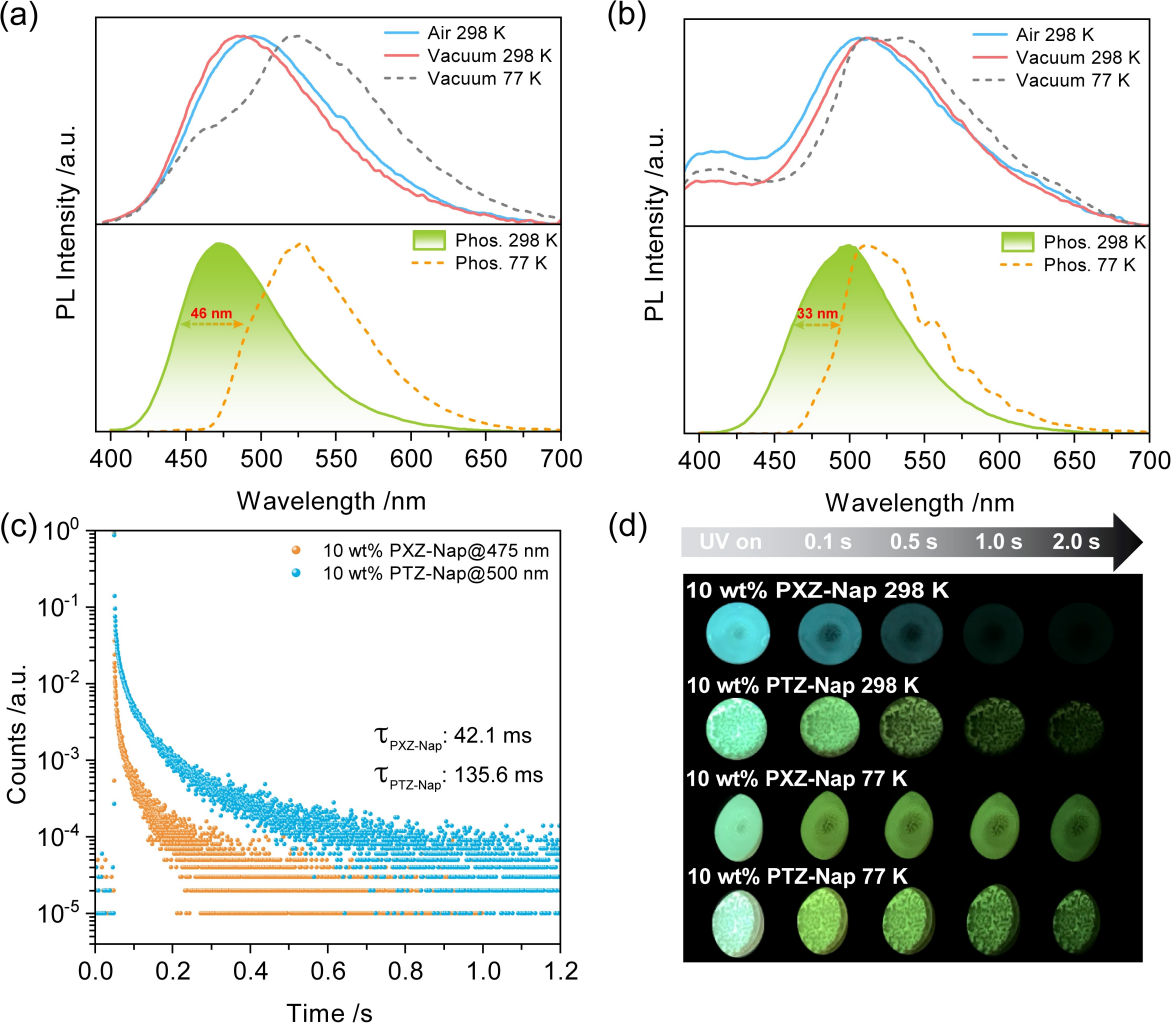
PL spectra of a) 10 wt % PXZ‐Nap and b) 10 wt % PTZ‐Nap at 298 K and 77 K; top: steady‐state emission spectra (*λ*_exc_=370 nm); bottom: phosphorescence spectra collected using MCS mode (*λ*_exc_=379 nm; time‐gated window: 10–200 ms for 298 K; 0.1–1.0 s for 77 K). c) RTP lifetime decay profiles of PXZ‐Nap and PTZ‐Nap. d) Images showing phosphorescence afterglows at 298 K and 77 K in vacuum (excitation source: 365 nm UV torch).

Considering aggregation can induce conformational changes,^[29]^ we next investigated the photophysics of the compounds at a higher concentration in PMMA films (10 wt %) to investigate the influence of intermolecular interactions on the phosphorescence behavior. Compared to the 1 wt % doped films of PXZ‐Nap and PTZ‐Nap in PMMA, a spectral red‐shift of the steady‐state PL occurs (Figures 7a and b). For PTZ‐Nap, the CT emission at around 505 nm is enhanced, associated with a 13.06 ns lifetime, compared with the LE emission at 400 nm (*τ*_PL_=5.02 ns) (Figure S24, Table S3). For PXZ‐Nap, the emergence of a blue‐shifted RTP compared to the broad CT fluorescence originates from thermal activation of triplet T_1_^L^ excitons to T_1_^H^ (Figure 7a). The 10 wt % film of PXZ‐Nap in PMMA exhibits similar RTP and LTP behavior to the 1 wt % doped film in PMMA, but with a shorter associated RTP lifetime (*τ*_Ph_=42.1 ms) (Figures 3c and 7c). Due to the similarity in RTP spectra with those of the 1 wt % doped films, it can be concluded that there is no additional contribution to the emission from aggregates. The distinct afterglows of the 10 wt % doped PMMA films of PXZ‐Nap at 298 K and 77 K also indicate emissions from two different T_1_ states (Figure 7d). Similarly, temperature‐dependent, and time‐gated‐dependent PL spectra also reveal the dual phosphorescence from T_1_^H^ and T_1_^L^ (Figures S25–S29).

At room temperature, the 10 wt % PTZ‐Nap doped PMMA film is more emissive than the 1 wt % PTZ‐Nap doped PMMA film, which is reflected also in the much longer RTP lifetime (Figure 7c) and the increased Φ_PL_ of the RTP (2.1 % vs. 0.3 %, Table S3). The LTP recorded at 77 K is more structured and is 33 nm red‐shifted compared with the RTP (Figure 7b). The temperature‐dependent delayed emission decay at 500 nm shows two distinct regimes. There is a shorter component that is thermally activated and that we assign to TADF (Figure S28), which likely results from RISC from T_1_^H^ to S_1_ as these two states are nearly degenerate, while the other long‐lived component (up to sub‐second) originates from phosphorescence. Compared with PXZ‐Nap, the improved Φ_PL_ (Table S3) of PTZ‐Nap is ascribed to the suppression of nonradiative decay processes. Based on these photophysical results, the intermolecular interactions present at higher doping concentrations are responsible for the modulation of the T_1_^H^ and T_1_^L^ emissions by influencing the T_1_ geometry.

**Figure 8:**
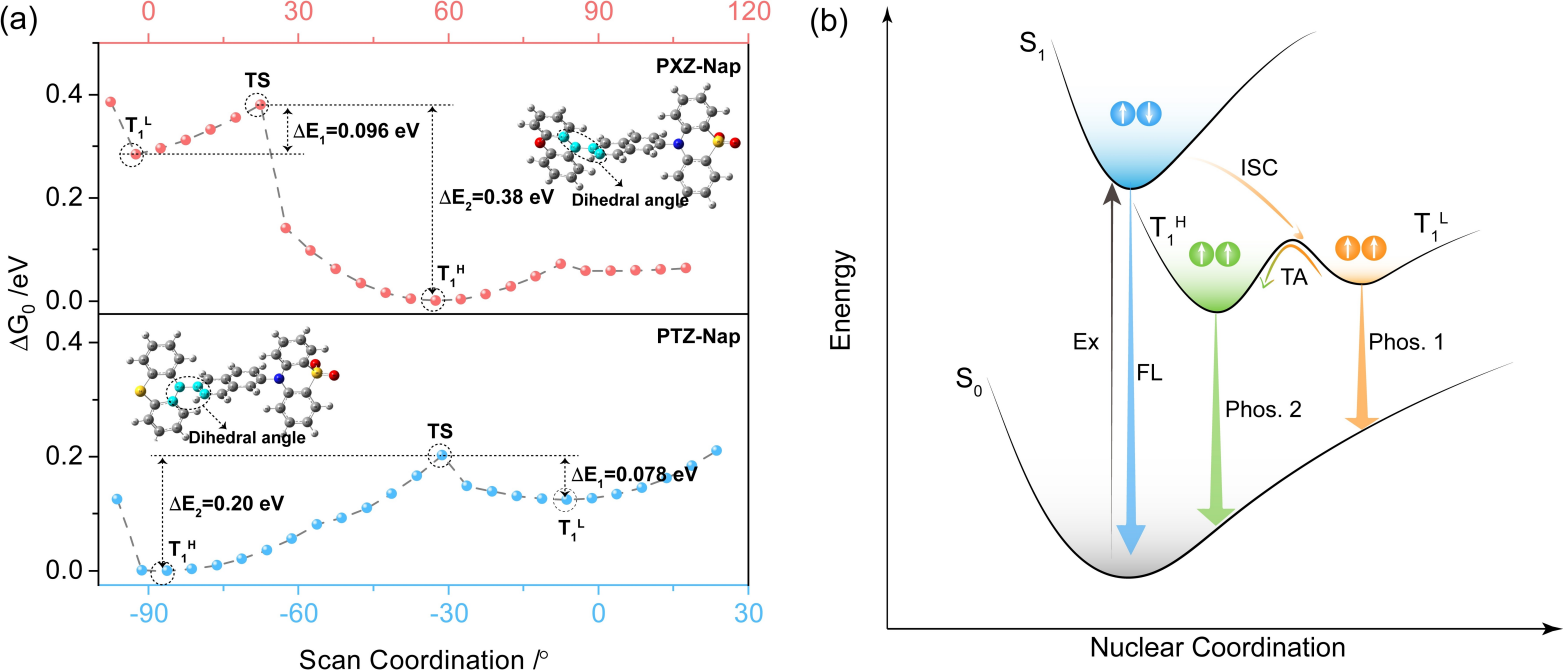
a) Potential energy surface scan of PXZ‐Nap (top) and PTZ‐Nap (bottom) calculated in the gas phase at the uPBE0/6‐31G(d,p) level. Scanned dihedral angle highlighted in cyan. b) Mechanistic illustration of conformation‐regulated high‐lying triplet exciton emission using a simplified Jablonski diagram. TA denotes thermal activation.

We next investigated how the conformational dynamics affects the T_1_ energy. A relaxed potential energy surface scan modelling for T_1_ conformations was conducted to rationalize the observed dual phosphorescence mechanism (Figure 8a). The dihedral angle between the donor and Nap was progressively modulated. From Figure 8a, the interconversion barrier (transition state, TS) between the two conformers is sufficiently small and thus the population of the T_1_^H^ conformer dominates. Thus, under photoexcitation, the populated singlet excitons intersystem cross and eventually relax to the lowest energy T_1_^L^ state (Figure 8b). At ambient temperature, the T_1_^L^ excitons can be thermally activated to access the T_1_^H^ and from this state RTP is observed. This process is suppressed at low temperature, resulting in the observed T_1_^L^ phosphorescence.

**Figure 9:**
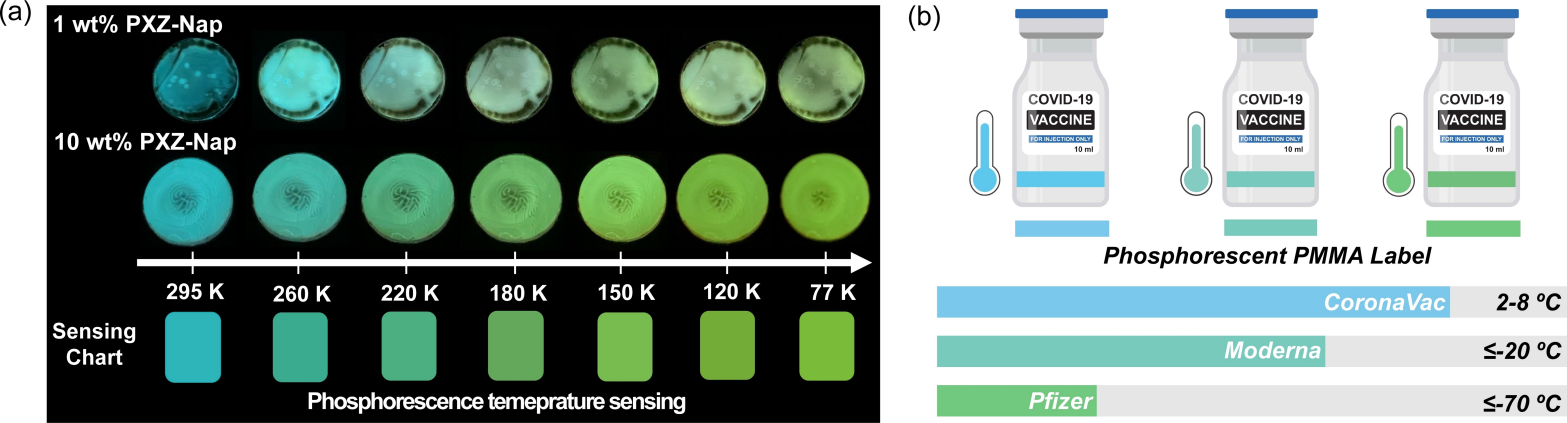
a) Temperature‐dependent phosphorescence afterglows of PXZ‐Nap in PMMA and fitted sensing charts. b) Designed COVID‐19 vaccine vials based on the phosphorescence PMMA label for monitoring the storage temperature.

Finally, benefitting from the significant difference in the spectral afterglow response to temperature of PXZ‐Nap doped in PMMA at 1 wt % and 10 wt %, these films were envisioned to act as temperature sensors. As shown in Figure 9a, the afterglow gradually varies from blue to green over the temperature range of 77–295 K. Considering most COVID‐19 vaccines must be stored below room temperature to be stable (Pfizer‐BioNTech COVID‐19: ≤−70 °C; Moderna COVID‐19 vaccine: ≤−20 °C; CoronaVac vaccine‐SARS‐CoV‐2: 2–8 °C), we demonstrate how our RTP system could be employed as a cost‐effective temperature sensor for monitoring the environment temperature in real time during the ultracold chain logistics and storage (Figure 9b).

## Conclusion

In summary, two RTP compounds (PXZ‐Nap and PTZ‐Nap) have been synthesized. Both compounds possess complex conformational dynamics in the excited state, evidenced by the observed dual RTP. In doped PMMA films, we demonstrated that RTP emission from the T_1_^H^ state originates from thermal activation between relaxed triplet excited state conformers. The photophysical studies in doped mCP films further support the observation that emitter‐matrix interactions can modulate triplet‐state emission. The Φ_RTP_ in mCP from T_1_^H^ was improved by a factor of 80.5 times to 16.1 % compared to the that in PMMA. We ascribe this enhancement due to the improvement of RTP efficiency as a result of Dexter energy transfer from the T_1_ of mCP to that of the guest emitters. Aggregation‐regulated triplet emission was demonstrated because of enhanced intermolecular interactions as observed in the photophysical behavior at higher doping concentration in PMMA. This study provides a rare yet clear picture of the triplet exciton dynamics in conformationally flexible donor–acceptor molecules and shows how RTP can be enhanced as a function of host‐emitter interactions. Given that we demonstrate that the relative populations of triplet excitons can be modulated as a function of the temperature, we demonstrated that these molecules could be used as functional temperature sensors for monitoring real‐time temperature for COVID‐19 vaccines during the ultracold chain logistics and storage.

## Supporting Information

^1^H and ^13^C‐NMR spectra, HRMS, EA of the target compounds; supplementary computational and photophysical data.

## Conflict of interest

The authors declare no competing financial interest.

1

## Supporting information

As a service to our authors and readers, this journal provides supporting information supplied by the authors. Such materials are peer reviewed and may be re‐organized for online delivery, but are not copy‐edited or typeset. Technical support issues arising from supporting information (other than missing files) should be addressed to the authors.

Supporting Information

Supporting Information

## Data Availability

The research data supporting this publication can be accessed at https://doi.org/10.17630/ffdb5242‐9f36‐4f68‐b782‐8ae240ab3896
