# Supplementary material for: Thermally Activated and Aggregation‐Regulated Excitonic Coupling Enable Emissive High‐Lying Triplet Excitons
Source: Angew Chem Int Ed Engl. 2022 Jul 4;61(33):e202206681. doi: 10.1002/anie.202206681 (PMC9545188; doi:10.1002/anie.202206681)
Supplement: Supplementary file 1 — Supporting Information [file ANIE-61-0-s001.pdf]

Angewandte Chemie
Eine Zeitschrift der Gesellschaft Deutscher Chemiker

Supporting Information

Thermally Activated and Aggregation-Regulated Excitonic Coupling Enable Emissive High-Lying Triplet Excitons

*T. Wang, J. De, S. Wu, A. K. Gupta, E. Zysman-Colman**

Table of Contents

General methods S3

Experimental section S7

Photophysical data S17

References S26

General Methods

General Synthetic Procedures

10*H*-phenothiazine 5,5-dioxide was synthesized according to a literature procedure.[1] All other reagents and solvents were obtained from commercial sources and used as received. Air-sensitive reactions were performed under a nitrogen atmosphere using Schlenk techniques, and no special precautions were taken to exclude air or moisture during work-up. Flash column chromatography was carried out using silica gel (Silica-P from Silicycle, 60 Å, 40-63 μm). Analytical thin-layer-chromatography (TLC) was performed with silica plates with aluminum backings (250 μm with F-254 indicator). TLC visualization was accomplished by a 254/365 nm UV lamp. ^{1}H and ^{13}C NMR spectra were recorded on a Bruker Advance spectrometer (500 MHz for ^{1}H, 125 MHz for ^{13}C) in d_6-DMSO and $CDCl_3$. The following abbreviations have been used for multiplicity assignments: "s" for singlet, "d" for doublet, "t" for triplet, "q" for quartet, "m" for multiplet. ^{1}H and ^{13}C NMR spectra were referenced residual solvent peaks with respect to TMS (δ = 0 ppm). Melting points were measured using open-ended capillaries on an Electrothermal 1101D Mel-Temp apparatus and are uncorrected. High-resolution mass spectrometry (HRMS) was performed at the University of Edinburgh. Elemental analyses were performed by the School of Geosciences at the University of Edinburgh.

Electrochemistry measurements

Cyclic Voltammetry (CV) analysis was performed on an Electrochemical Analyzer potentiostat model 620E from CH Instruments at a sweep rate of 100 mV/s. Differential pulse voltammetry (DPV) was conducted with an increment potential of 0.01 V and a pulse amplitude, width, and period of 50 mV, 0.06, and 0.5 s, respectively. Samples (**PXZ-Nap** and **PTZ-Nap**) were prepared as *N, N*-dimethylformamide solutions, degassed by sparging with DMF-saturated N_2 for 5 minutes before measurements. All measurements were performed using 0.1 M DMF solution of tetra-*n*-butylammonium

hexafluorophosphate ([nBu$_4$N]PF$_6$]). An Ag/Ag$^+$ electrode was used as the reference electrode, while a glassy carbon electrode and a platinum wire were used as the working electrode and counter electrode, respectively. The redox potentials are reported relative to a saturated calomel electrode (SCE) with a ferrocenium/ferrocene (Fc/Fc$^+$) redox couple as the internal standard (0.45 V vs. SCE).[2] The HOMO and LUMO energies were determined using the relation $E_{HOMO/LUMO} = -(E_{ox} / E_{red} + 4.8)$ eV, where E_{ox} and E_{red} are the onset of anodic and cathodic peak potentials, respectively calculated from DPV relative to Fc/Fc$^+$.[3]

Theoretical calculations

All ground state optimizations have been carried out at the Density Functional Theory (DFT) level with Gaussian 16[4] using the PBE0 functional[5] and the 6-31G(d,p) basis set.[6] Vibrational frequency calculations were performed to ensure that the optimized geometries represented the local minima. Excited-state calculations have been performed at time-dependent DFT (TD-DFT) using the same functional and basis set as for ground state geometry optimization. Spin-orbit coupling matrix elements (ξ) were calculated based on the optimized singlet excited state geometry. Triplet excited state optimizations were conducted at the uPBE0/6-31G(d,p) level. Potential surface energy scan was conducted using optimized triple excited-state geometry. Molecular orbitals were visualized using GaussView 6.0.[7] Spin-orbit coupling matrix elements between singlet and triplet excited states were calculated using the PySOC program.[8]

Photophysical measurements

Optically dilute solutions of concentrations on the order of 10^{-5} or 10^{-6} M were prepared in spectroscopic or HPLC grade solvents for absorption and emission analysis. Absorption spectra were recorded at room temperature on a Shimazu UV-2600 double beam spectrophotometer with a 1 cm quartz cuvette. Molar absorptivity determination was verified by linear regression analysis of values obtained from at least four independent solutions at varying concentrations with absorbance ranging from 3.27 $\times 10^{-6}$ to 2.12 $\times 10^{-5}$ M.

For emission investigations, degassed solutions were prepared via three freeze-pump-thaw cycles, and spectra were taken using homemade Schlenk quartz cuvette. Steady-state and time-resolved emission spectra were recorded at 298 K using an Edinburgh Instruments F980. Samples were excited at 370 nm for steady-state measurements and at 379 nm (picosecond laser) for time-resolved measurements. Phosphorescence emission spectra were collected with a 379 nm picosecond laser by the multi-channel scaling (MCS) mode.

Photoluminescence quantum yields for solutions were determined using the optically dilute method[9] in which four sample solutions with absorbances of ca. 0.10, 0.075, 0.050 and 0.025 at 360 nm were used. The Beer-Lambert law was found to remain linear at the concentrations of the solutions. For each sample, linearity between absorption and emission intensity was verified through linear regression analysis with the Pearson regression factor (R^2) for the linear fit of the data set surpassing 0.9. Individual relative quantum yield values were calculated for each solution and the values reported represent the slope obtained from the linear fit of these results. The quantum yield of the sample, Φ_{PL}, can be determined by the equation $\Phi_{PL} = (\Phi_r * \frac{A_r}{A_s} * \frac{I_s}{I_r} * \frac{n_s^2}{n_r^2})$,[9] where A stands for the absorbance at the excitation wavelength (λ_{exc}: 370 nm), I is the integrated area under the corrected emission curve and n is the refractive index of the solvent with the subscripts "s" and "r" representing sample and reference respectively. Φ_r is the absolute quantum yield of the external reference quinine sulfate (Φ_r = 54.6% in 1 *N* H_2SO_4),[10] The experimental uncertainty in the emission quantum yields is conservatively estimated to be 10%, though we have found that statistically we can reproduce Φ_{PL}s to 3% relative error. An integrating sphere (Hamamatsu, C9920-02) was employed for the photoluminescence quantum yield measurements of thin film samples. The Φ_{PL} of the films were then measured in air and N_2 environment by purging the integrating sphere with N_2 gas flow.

The singlet-triplet energy splitting (ΔE_{ST}) in 2-MeTHF was estimated by recording the prompt fluorescence spectra and phosphorescence emission at 77 K. The films were excited by a femtosecond laser emitting at 343 nm (Orpheus-N, model: SP-06-200-PP).

Emission from the samples was focused onto a spectrograph (Chromex imaging, 250is spectrograph) and detected on a sensitive gated iCCD camera (Stanford Computer Optics, 4Picos) having subnanosecond resolution. Phosphorescence spectra were measured from 1 ms after photoexcitation, with an iCCD exposure time was 7.5 ms. Prompt fluorescence spectra were measured from 1 ns after photoexcitation with an iCCD exposure time was 99 ns.

Fitting of time-resolved luminescence measurements

Time-resolved PL measurements were fitted to a sum of exponential decay model, with chi-squared (χ^2) values between 1 and 2, using the EI FLS980 software. Each component of the decay is assigned a weight, (w_i), which is the contribution of the emission from each component to the total emission.

The average lifetime was then calculated using the following:

- Two exponential decay model:

$$\tau_{AVG} = \tau_1 w_1 + \tau_2 w_2$$

with weights defined as $w_1 = \frac{A1\tau_1}{A1\tau_1 + A2\tau_2}$ and $w_2 = \frac{A2\tau_2}{A1\tau_1 + A2\tau_2}$ where A1 and A2 are the preexponential-factors of each component.

- Three exponential decay model:

$$\tau_{AVG} = \tau_1 w_1 + \tau_2 w_2 + \tau_3 w_3$$

with weights defined as $w_1 = \frac{A1\tau_1}{A1\tau_1 + A2\tau_2 + A3\tau_3}$, $w_2 = \frac{A2\tau_2}{A1\tau_1 + A2\tau_2 + A3\tau_3}$ and $w_3 = \frac{A3\tau_3}{A1\tau_1 + A2\tau_2 + A3\tau_3}$ where A1, A2 and A3 are the preexponential-factors of each component.

Experimental Section

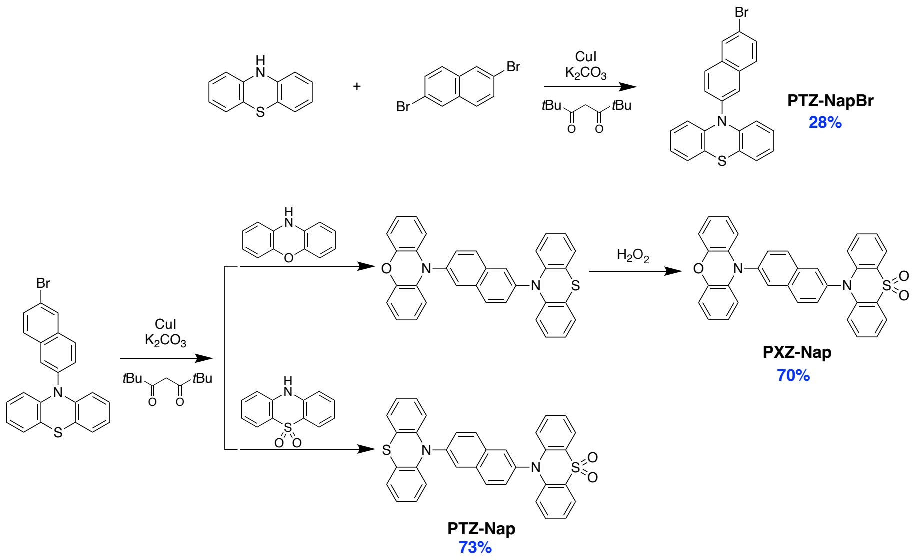

Scheme S1. Synthetic Routes and chemical structures of **PTZ-NapBr**, **PXZ-Nap,** and **PTZ-Nap**

Synthesis of 10-(6-bromonaphthalen-2-yl)-10H-phenothiazine (PTZ-NapBr)

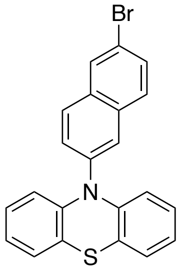

Phenothiazine (2.00 g, 10.0 mmol, 1.0 equiv.), 2,6-dibromonapthalene (4.30 g, 15.0 mmol, 1.5 equiv.), potassium carbonate (2.76 g, 20.0 mmol, 2 equiv.), copper iodide (0.19 g, 1.0 mmol, 0.1 equiv.), and 2,2,6,6-tetramethylheptane-3,5-dione (0.37 g, 2.0 mmol, 0.2 equiv.) were added to a Schlenk flask containing 50 mL of anhydrous DMF. After degassing the flask, the system was placed under a nitrogen atmosphere. The reaction mixture was heated at 150 °C for 36 h. After cooling to room temperature, DCM (200 mL) was added to the mixture. The mixture was washed with a saturated NaCl aqueous solution (200 mL) and further with a 1 M HCl solution (2 × 200 mL). The collected organic phase was dried over anhydrous sodium sulfate and concentrated under reduced pressure. The crude mixture was purified by

silica gel column chromatography (DCM: Hexane = 1:4, **R_f**: 0.23) to afford the desired compound as a white solid. **Yield**: 1.14 g, 28%. **Mp**: 168-169 °C. **^{1}H NMR (500 MHz, d_6-DMSO) δ (ppm)**: 8.38 (d, J = 2.1 Hz, 1H), 8.20 (d, J = 8.7 Hz, 1H), 8.09 (d, J = 2.1 Hz, 1H), 8.00 (d, J = 8.8 Hz, 1H), 7.73 (dd, J = 8.7, 2.0 Hz, 1H), 7.60 (dd, J = 8.7, 2.1 Hz, 1H), 7.18 – 7.11 (m, 2H), 6.97 – 6.87 (m, 4H), 6.28 – 6.20 (m, 2H). **^{13}C NMR (125 MHz, d_6-DMSO) δ (ppm)**: 143.94, 138.93, 133.86, 133.47, 130.68, 130.57, 130.19, 130.13, 129.67, 128.83, 127.87, 127.27, 123.46, 120.56, 120.38, 117.07. GC-MS (m/z): 403.05. The characterization matches the reported results.[11]

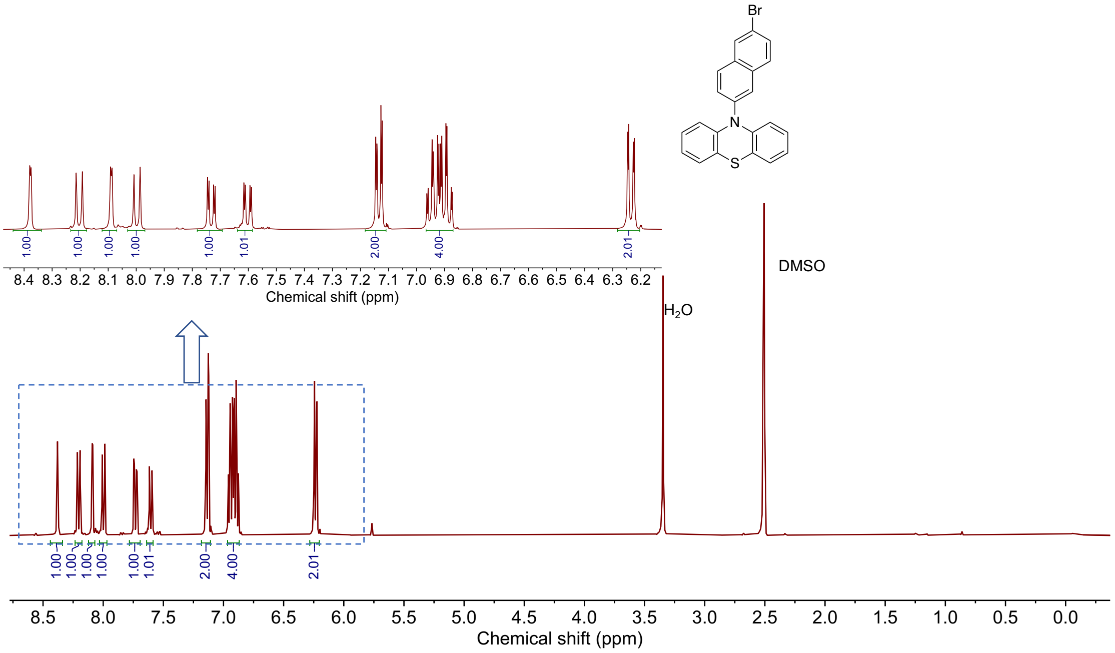

Figure S1. ^{1}H NMR spectrum of **PTZ-NapBr** in d_6-DMSO.

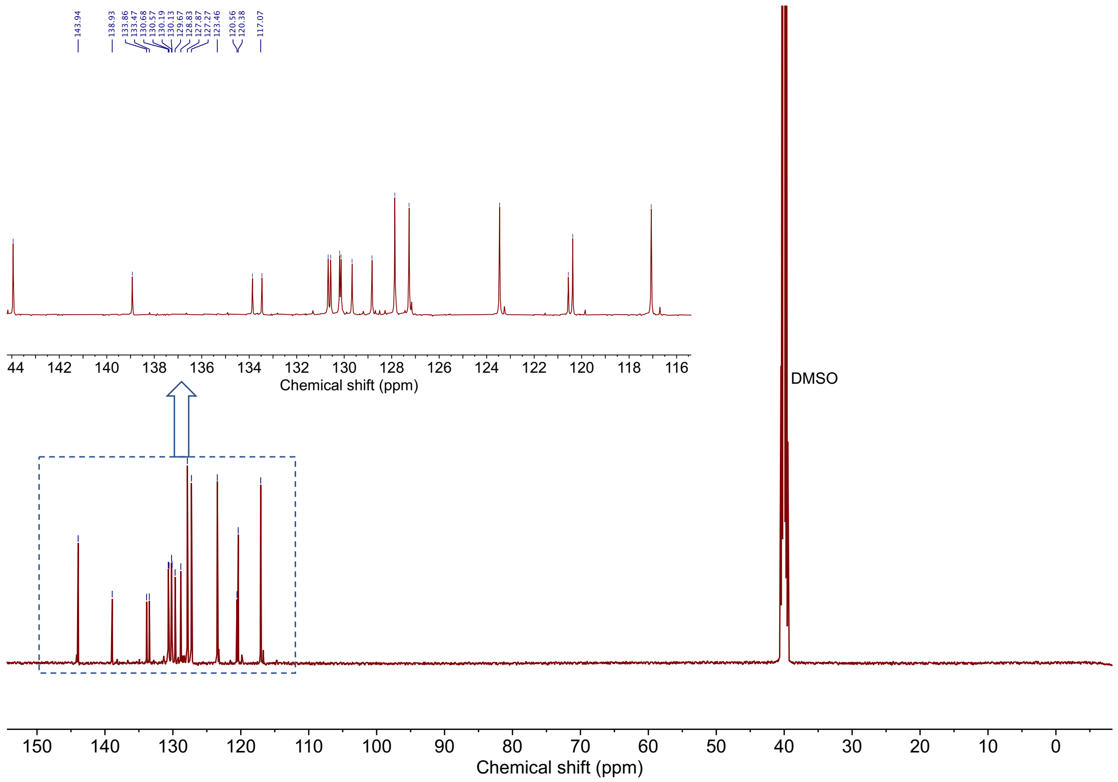

Figure S2. ^{13}C NMR spectrum of **PTZ-NapBr** in d_6-DMSO.

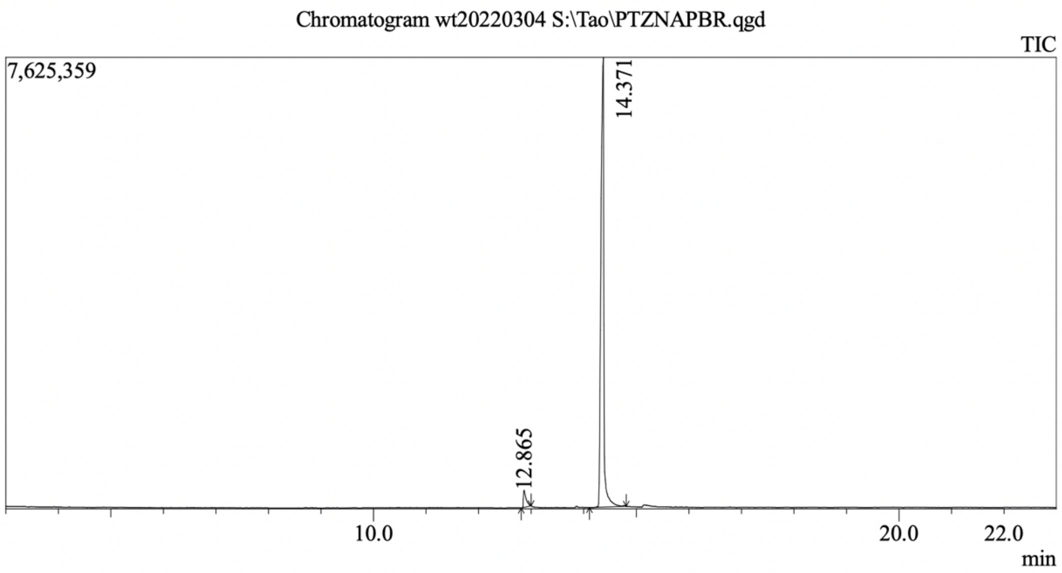

Peak Report TIC

Peak#	R.Time	Area	Area%	Height	A/H	Base m/z	Base Int.
1	12.865	1042582	3.40	287588	3.63	325.15	77789
2	14.371	29619799	96.60	7593889	3.90	403.05	846874
		30662381	100.00	7881477			

Figure S3. GC-MS trace of **PTZ-NapBr**.

Synthesis of 10-(6-(10*H*-phenoxazin-10-yl)naphthalen-2-yl)-10*H*-phenothiazine 5,5-dioxide (PXZ-Nap)

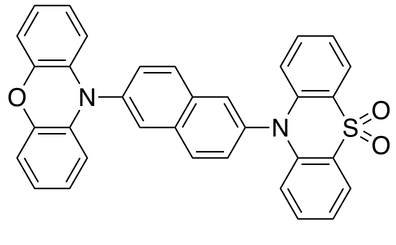

Phenoxazine (0.41 g, 2.23 mmol, 1.5 equiv.), **PTZ-NapBr** (0.60 g, 1.45 mmol, 1.0 equiv.), potassium carbonate (0.41 g, 2.97 mmol, 2.0 equiv.), copper iodide (0.029 g, 0.15 mmol, 0.1 equiv.), and 2,2,6,6-tetramethylheptane-3,5-dione (0.055 g, 0.30 mmol, 0.2 equiv.) were added to a Schlenk flask containing 6 mL of anhydrous DMF. After degassing the flask, the system was placed under a nitrogen atmosphere. The reaction mixture was heated at 150 °C for 36 h. After cooling to room temperature, DCM (100 mL) was added to the mixture. The mixture was washed with a saturated NaCl aqueous solution (100 mL) and further with a 1M HCl solution (2 × 100 mL). The collected organic phase was dried over anhydrous sodium sulfate and concentrated under reduced pressure. The crude mixture was dissolved in acetic acid (8 mL) and 30% hydrogen peroxide (1 mL) was added. The mixture system was heated at 120 °C 10 hours. After cooling, 40 mL H_2O was added to the system. The collected precipitates were purified by silica gel column chromatography (EtOAc: Hexane = 1:2, **R*f***: 0.26) to afford the desired compound as a light-yellow solid. **Yield**: 0.54 g, 70%. **Mp**: >390 °C. **^{1}H NMR (500 MHz, *d*$_6$-DMSO) δ (ppm)**: 8.45 – 8.32 (m, 4H), 8.14 (dd, J = 7.9, 1.6 Hz, 2H), 7.68 (ddd, J = 18.5, 8.6, 2.1 Hz, 2H), 7.58 (ddd, J = 8.8, 7.2, 1.6 Hz, 2H), 7.39 (ddd, J = 8.0, 7.1, 1.0 Hz, 2H), 6.81 (dd, J = 7.7, 1.7 Hz, 2H), 6.75 – 6.68 (m, 6H), 6.00 (dd, J = 7.7, 1.7 Hz, 2H). **^{13}C NMR (125 MHz, *d*$_6$-DMSO) δ (ppm)**: 143.66, 140.71, 137.73, 137.09, 134.98, 134.39, 134.24, 134.05, 132.60, 132.28, 130.50, 130.00, 129.43, 128.64, 124.28, 123.32, 122.92, 122.56, 122.22, 118.01, 115.93, 113.97. **HR-MS $[M+H]^+$ Calculated**: ($C_{34}H_{22}N_2O_3S$) 539.1424; **Found**: 539.1401. **Anal. Calcd. for $C_{34}H_{22}N_2O_3S$**: C, 75.82%; H, 4.12%; N, 5.20%. **Found**: C, 76.45%; H, 4.03%; N, 4.92%. Purity: 99.90% (HPLC analysis; retention time: 6.95 minutes in 75% MeCN/25% H_2O).

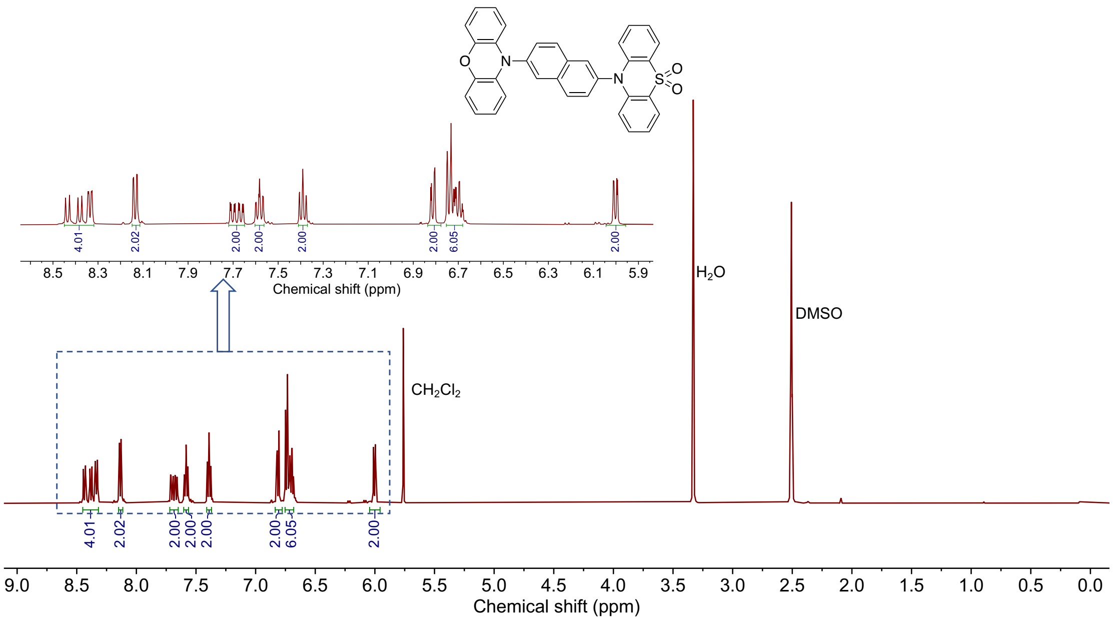

Figure S4. ^{1}H NMR spectrum of **PXZ-Nap** in d_6-DMSO.

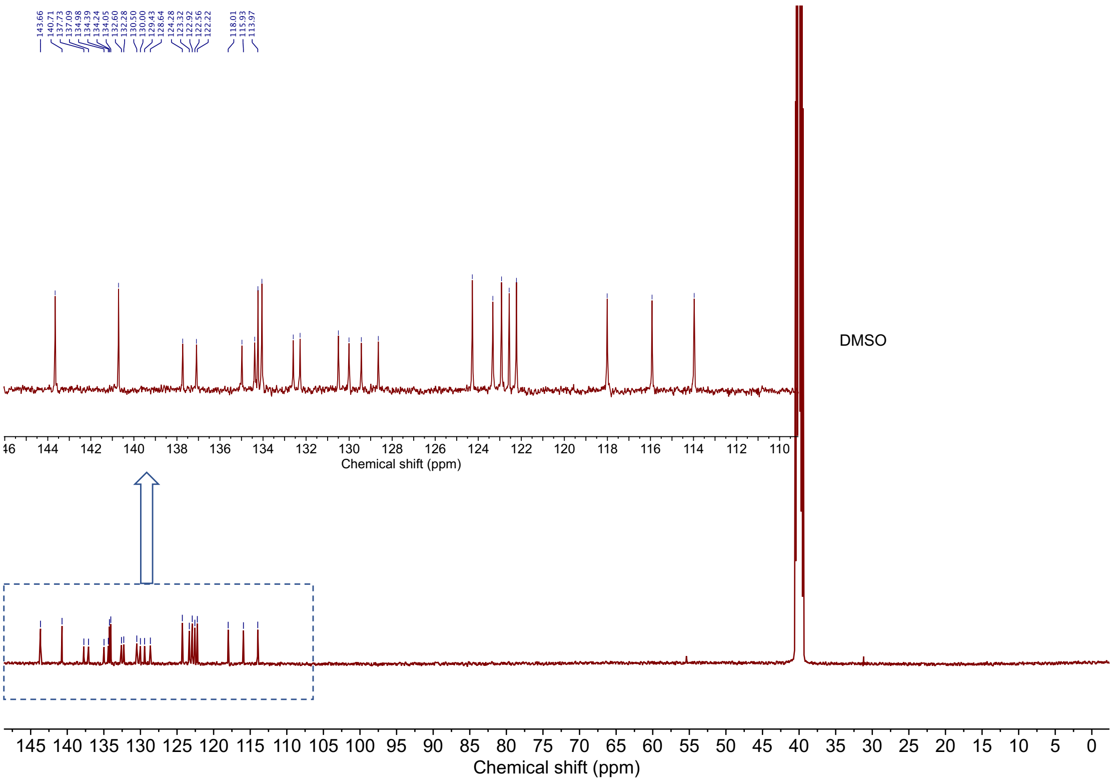

Figure S5. ^{13}C NMR spectrum of **PXZ-Nap** in d_6-DMSO.

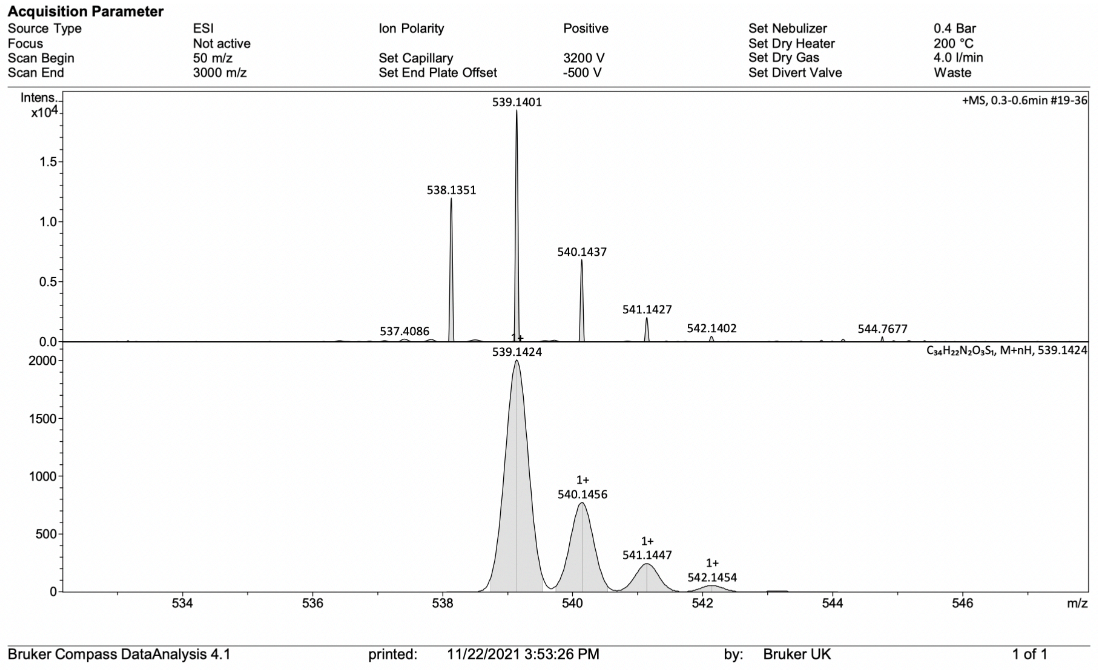

Figure S6. HRMS spectrum of **PXZ-Nap**.

Analysis type:

Single ☐ **Duplicate** ☑ **Triplicate** ☐

Analysis Result:

Element	Expected %	Found (1)	Found (2)	Found (3)
Carbon	75.82	76.45	75.02	
Hydrogen	4.12	4.03	4.11	
Nitrogen	5.20	4.92	4.85	
Oxygen				

Authorising Signature:

Date completed	15.12.21
Signature	J -PC
comments	

Figure S7. Elemental analysis result of **PXZ-Nap**.

HPLC Trace Report14Feb2022

<Sample Information>

Sample Name	: PXZ-Nap		
Sample ID	:		
Method Filename	: 75% Acetonitrile 25 Water 20 mins.lcm		
Batch Filename	: PhT-Nap method.lcb		
Vial #	: 2-48	Sample Type	: Unknown
Injection Volume	: 10 uL		
Date Acquired	: 14/02/2022 19:44:16	Acquired by	: System Administrator
Date Processed	: 14/02/2022 20:04:19	Processed by	: System Administrator

<Chromatogram>

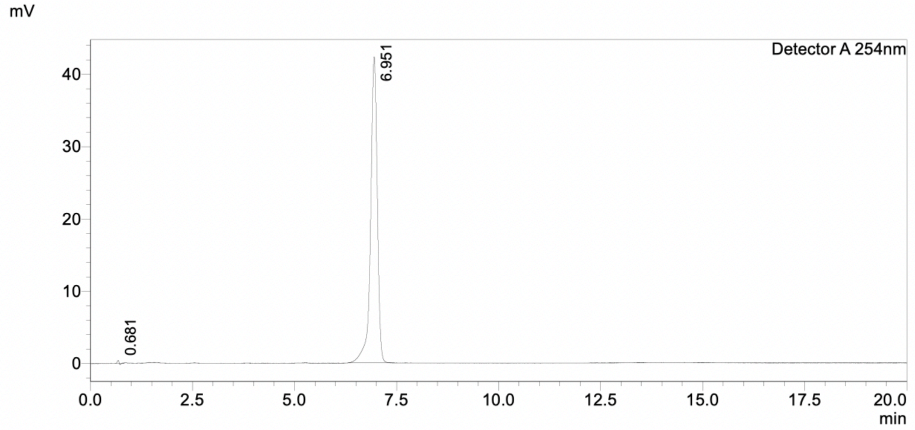

<Peak Table>

Detector A 254nm

Peak#	Ret. Time	Area	Height	Area%	Area/Height	Width at 5% Height
1	0.681	486	395	0.100	1.228	0.064
2	6.951	486058	42258	99.900	11.502	0.444
Total		486544	42653	100.000		

Figure S8. HPLC trace of **PXZ-Nap**. Note: at the retention time of 0.68 min, the peak is the background signal from the elution system because the sample signal is not enough strong caused by the solubility.

Synthesis of 10-(6-(10*H*-phenothiazin-10-yl)naphthalen-2-yl)-10*H*-phenothiazine 5,5-dioxide (PTZ-Nap)

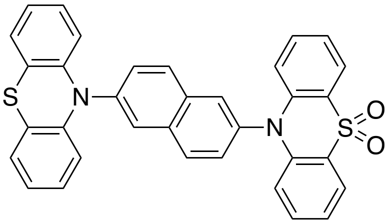

10*H*-phenothiazine 5,5-dioxide (0.43 g, 1.85 mmol, 1.5 equiv.), **PTZ-NapBr** (0.50 g, 1.24 mmol, 1.0 equiv.), potassium carbonate (0.41 g, 2.47 mmol, 2.0 equiv.), copper iodide (0.041 g, 0.12 mmol, 0.1 equiv.), and 2,2,6,6-tetramethylheptane-3,5-dione (0.055 g, 0.24 mmol, 0.2 equiv.) were added to a Schlenk flask containing 6 mL of anhydrous DMF. After degassing the flask, the system was placed under a nitrogen atmosphere. The reaction mixture was heated at 150 °C for 36 h. After cooling to room

temperature, DCM (100 mL) was added to the mixture. The mixture was washed with a saturated NaCl aqueous solution (100 mL) and further with a 1M HCl solution (2 × 100 mL). The collected organic phase was dried over anhydrous sodium sulfate and concentrated under reduced pressure. The crude mixture was purified by silica gel column chromatography (EtOAc: Hexane = 1:2, **R*f***: 0.24) to afford the desired compound as a light-yellow solid. **Yield**: 0.50 g, 73%. **Mp**: >390 °C. **^{1}H NMR (500 MHz, $CDCl_3$) δ (ppm)**: 8.24 (dd, J = 7.9, 1.6 Hz, 2H), 8.19 (d, J = 8.6 Hz, 1H), 8.13 (d, J = 8.7 Hz, 1H), 8.04 (t, J = 2.3 Hz, 2H), 7.64 (dd, J = 8.7, 2.1 Hz, 1H), 7.51 (dd, J = 8.6, 2.0 Hz, 1H), 7.42 (ddd, J = 8.8, 7.1, 1.7 Hz, 2H), 7.32 – 7.29 (m, 2H), 7.22 – 7.14 (m, 2H), 7.01 – 6.92 (m, 4H), 6.73 (dd, J = 8.7, 0.9 Hz, 2H), 6.52 – 6.45 (m, 2H). **NMR (125 MHz, $CDCl_3$) δ (ppm)**: 143.84, 141.04, 140.89, 136.64, 134.76, 133.34, 132.84, 131.46, 130.76, 129.71, 128.81, 128.24, 127.27, 127.09, 126.99, 123.54, 123.33, 122.81, 122.50, 122.23, 117.70, 117.31. **HR-MS [M+H]$^+$ Calculated**: ($C_{34}H_{22}N_2O_2S_2$) 555.1195; **Found**: 555.1195. **Anal. Calcd. for $C_{34}H_{22}N_2O_2S_2$**: C, 73.62%; H, 4.00%; N, 5.05%. **Found**: C, 73.60%; H, 4.07%; N, 4.72%. Purity: 99.4% (HPLC analysis; retention time: 7.75 minutes in 75% MeCN/25% H_2O).

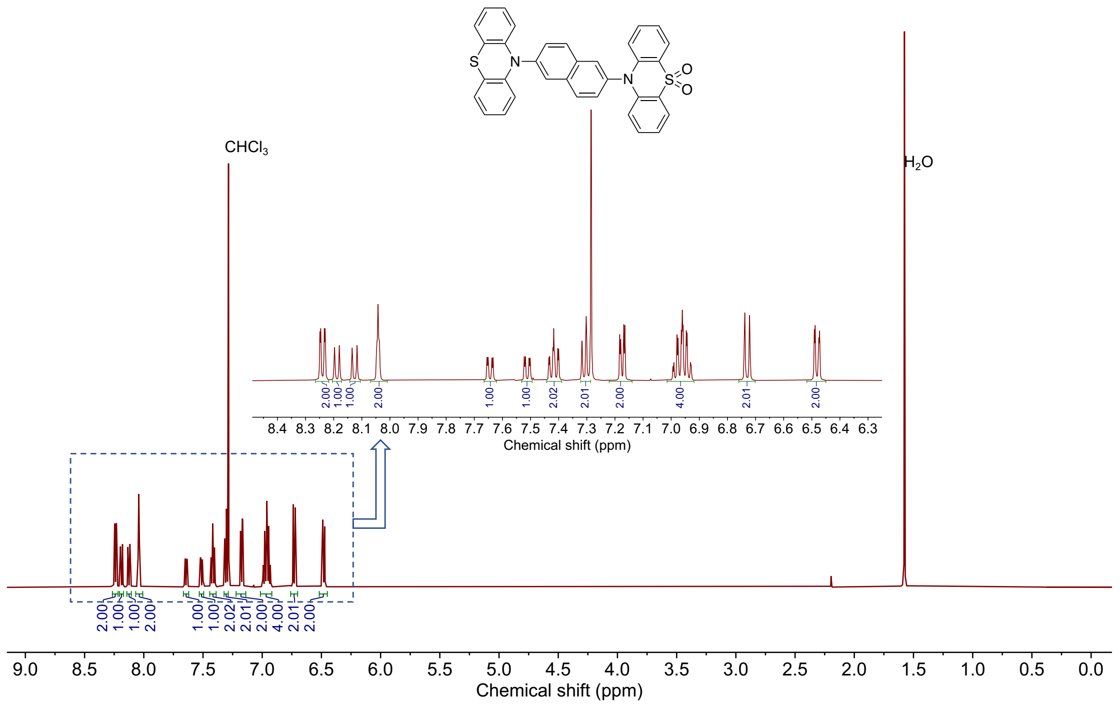

Figure S9. ^{1}H NMR spectrum of **PTZ-Nap** in $CDCl_3$.

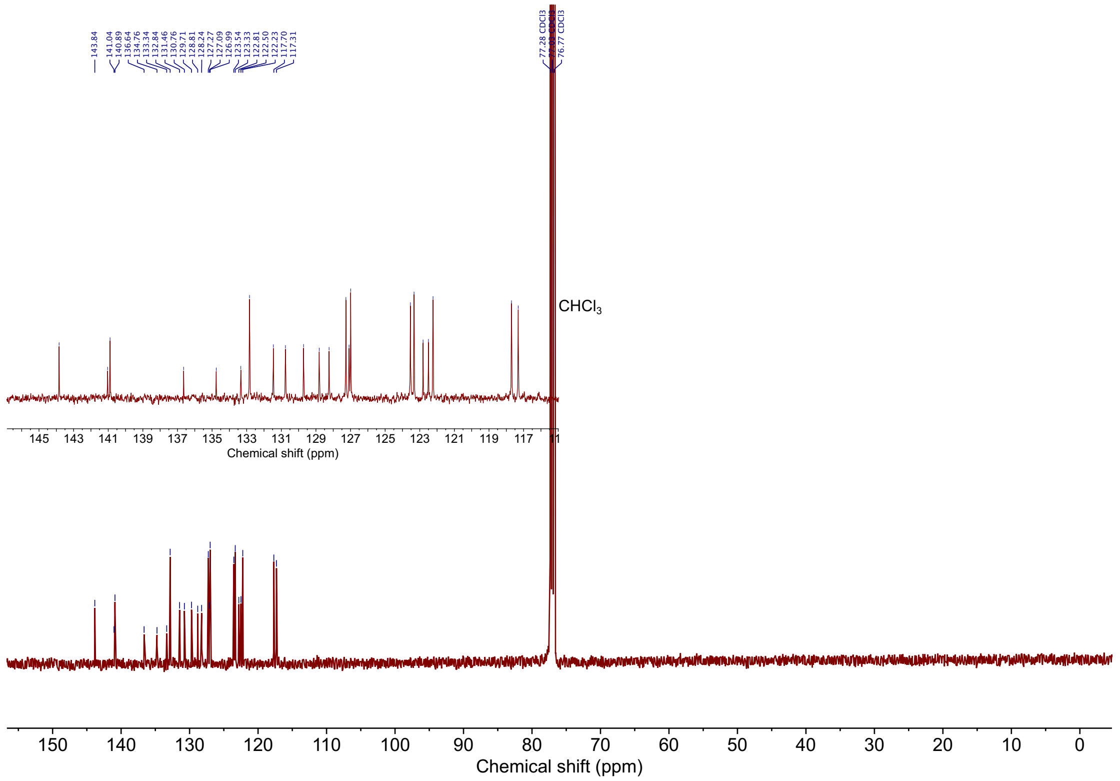

Figure S10. ^{13}C NMR spectrum of **PTZ-Nap** in $CDCl_3$.

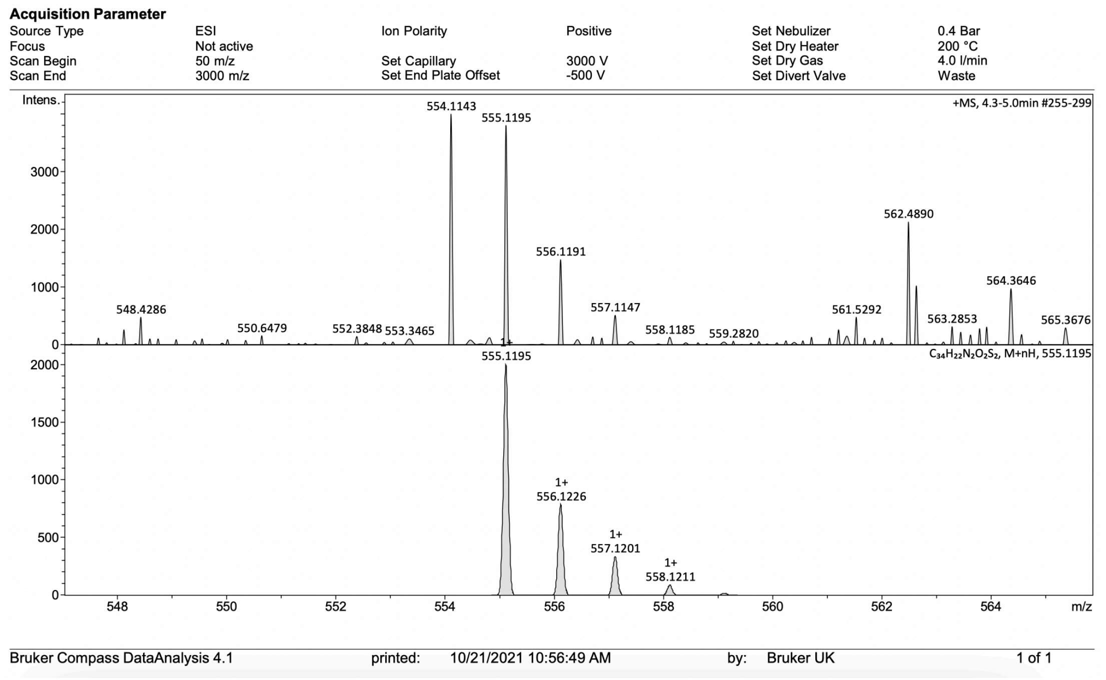

Figure S11. HRMS spectrum of **PTZ-Nap**.

Analysis type:

Single ☐ **Duplicate** ☑ **Triplicate** ☐

Analysis Result:

Element	Expected %	Found (1)	Found (2)	Found (3)
Carbon	73.62	73.38	73.60	
Hydrogen	4.00	4.05	4.07	
Nitrogen	5.05	4.70	4.72	
Oxygen				

Authorising Signature:

**Date completed**	15.12.21
**Signature**	J-PC
**comments**	

Figure S12. Elemental analysis result of **PTZ-Nap**.

HPLC Trace Report04Mar2022

<Sample Information>

Sample Name	: PhT-Nap		
Sample ID	:		
Method Filename	: 75% Acetonitrile 25 Water 20 mins.lcm		
Batch Filename	: PhT-Nap method.lcb		
Vial #	: 2-47	Sample Type	: Unknown
Injection Volume	: 10 uL		
Date Acquired	: 14/02/2022 18:20:26	Acquired by	: System Administrator
Date Processed	: 14/02/2022 18:40:28	Processed by	: System Administrator

<Chromatogram>

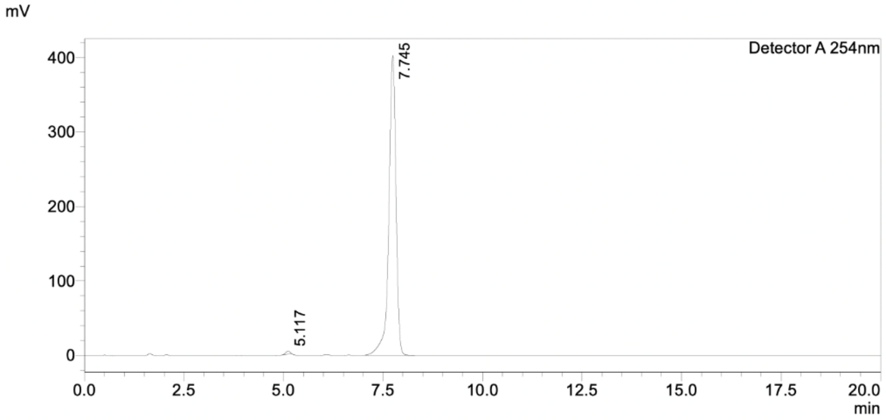

<Peak Table>

Detector A 254nm

Peak#	Ret. Time	Area	Height	Area%	Area/Height	Width at 5% Height
1	5.117	30673	4168	0.606	7.359	0.209
2	7.745	5033444	401997	99.394	12.521	0.476
Total		5064117	406165	100.000		

Figure S13. HPLC trace of **PTZ-Nap**.

Photophysical data

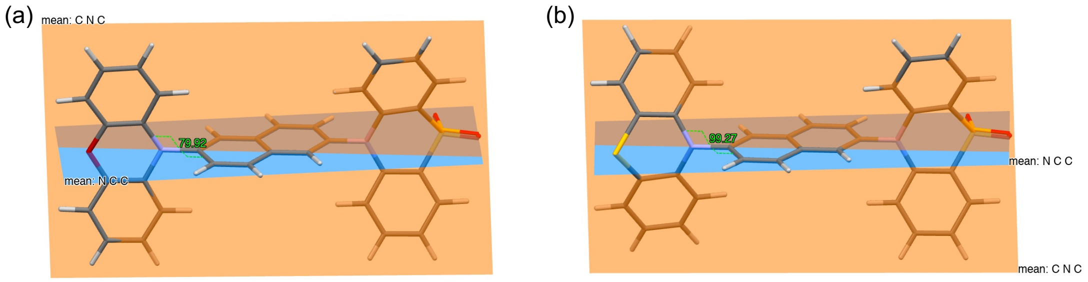

Figure S14. Optimized ground state structures of (a) **PXZ-Nap** and (b) **PTZ-Nap** was conducted at the PBE0/6-31G(d,p) level in the gas phase.

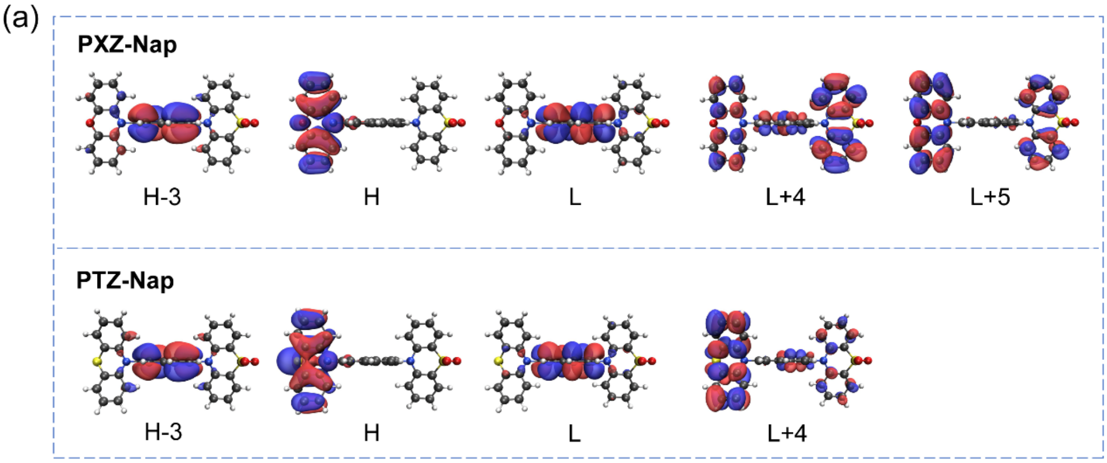

(b)

PXZ-Nap

State	Transition (%)	ΔE_{S1T1} $\langle S_1 \vert H_{SO} \vert T_1 \rangle$
S_1 2.57 eV	H→L 98	0.05 eV<br>0.09 cm^{-1}
T_1 2.52 eV	H→L 92	
T_2 2.60 eV	H-3→L 85	
T_3 2.88 eV	H→L+4 55<br>H→L+5 18	

PTZ-Nap

State	Transition (%)	ΔE_{S1T1} $\langle S_1 \vert H_{SO} \vert T_1 \rangle$
S_1 2.80 eV	H→L 97	0.24 eV<br>0.12 cm^{-1}
T_1 2.56 eV	H-3→L 87	
T_2 2.73 eV	H→L 84<br>H→L+4 10	
T_3 3.03 eV	H→L+4 55<br>H→L 13	

Figure S15. (a) Frontier molecular orbitals (isovalue: 0.02) and (b) excited-state information of **PXZ-Nap** and **PTZ-Nap** calculated in the gas phase at the PBE0/6-31G(d,p) level. H and L denote HOMO and LUMO, respectively.

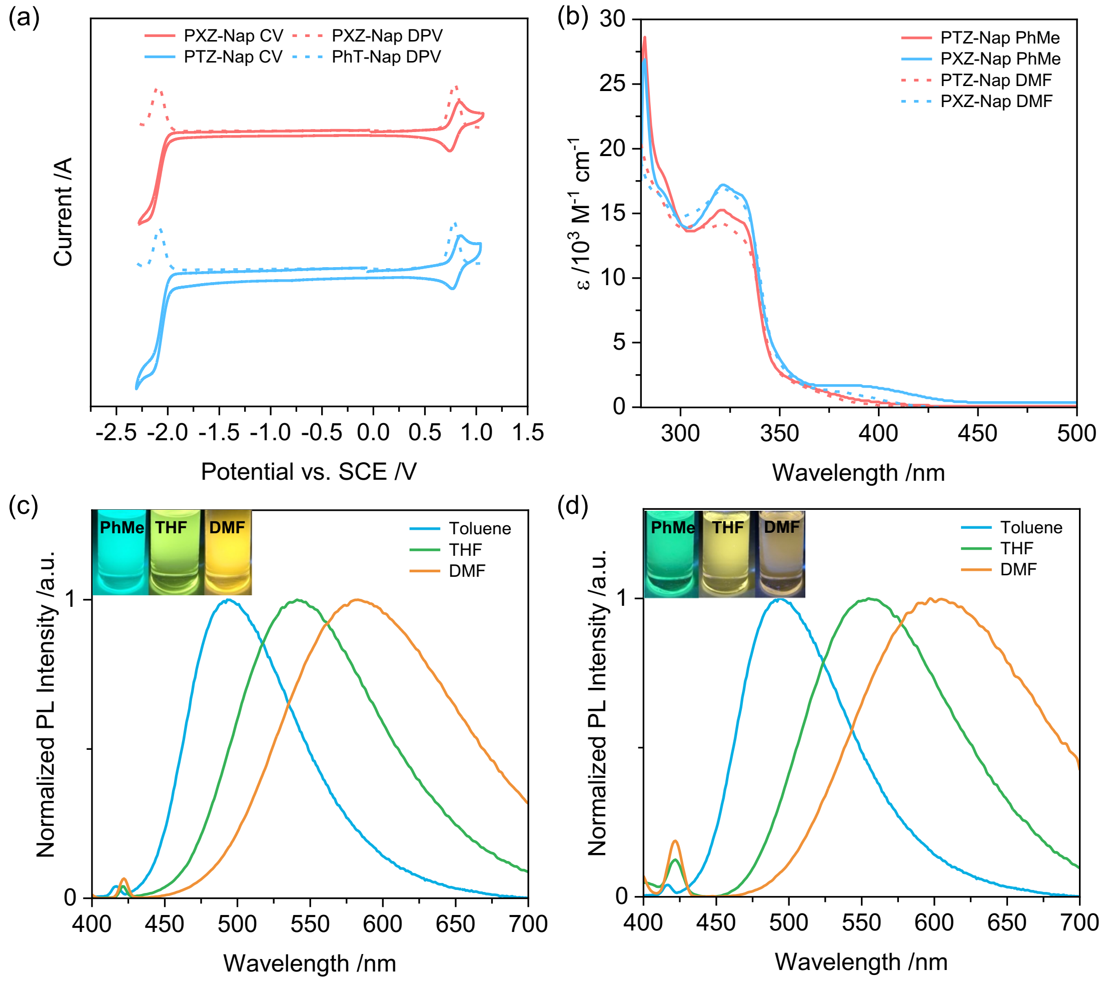

Figure S16. (a) Cyclic voltammogram (CV) and differential pulse voltammetry (DPV) in degassed DMF with 0.1 M [*n*Bu$_4$N]PF$_6$ as the supporting electrolyte and Fc/Fc$^+$ as the internal reference (0.45 V vs. SCE).[2] $E_{HOMO/LUMO}$= -(E^{ox}/E^{red} + 4.8) eV, using Fc/Fc$^+$ as the internal reference.[3] (b) UV-vis absorption spectra in toluene and DMF at room temperature. Steady-state solvatochromic PL spectra of (c) **PXZ-Nap** and (d) **PTZ-Nap** at room temperature (λ_{exc} = 370 nm); inset: photos of solution samples excited using a UV torch.

Table S1. Electrochemical data of **PXZ-Nap** and **PTZ-Nap**.

	E^{ox} / V[a]	E^{red}/ V[a]	HOMO / eV[b]	LUMO / eV[b]
**PXZ-Nap**	0.79	-2.09	-5.14	-2.26
**PTZ-Nap**	0.78	-2.09	-5.13	-2.26

[a]Potential values were obtained from the DPV peak values in DMF and referenced with respect to SCE (Fc/Fc^+ = 0.45 eV).[2] [b]$E_{HOMO/LUMO}$= -(E^{ox}/E^{red} + 4.8) eV, using Fc/Fc^+ as the internal reference.[3]

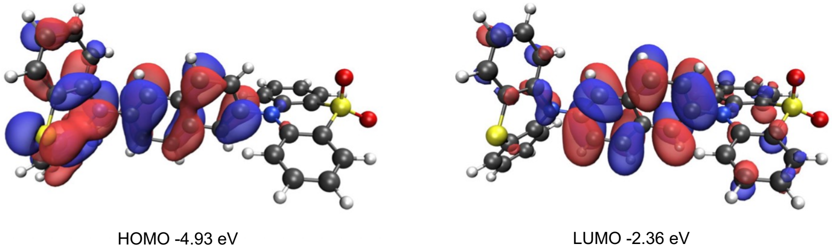

Figure S17. Calculated HOMO and LUMO distribution (isovalue: 0.02) of the quasi-axial conformation of **PTZ-Nap** at the PBE0/6-31G(d,p) level in DMF. We can clearly see that HOMO is delocalized on **PTZ** and naphthalene due to the quasi-axial conformation in DMF.

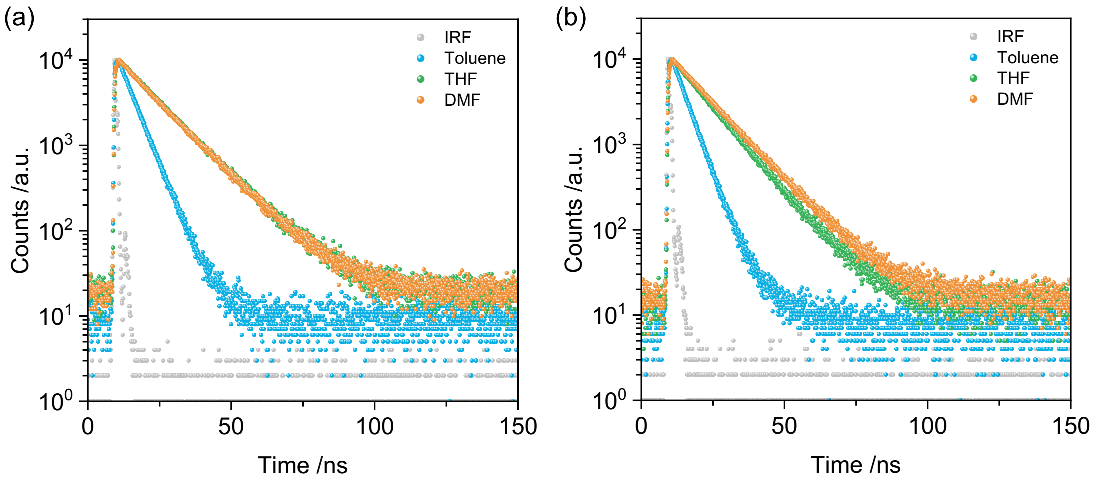

Figure S18. Time-resolved PL decay profiles of (a) **PXZ-Nap** and **PTZ-Nap** in various solvents at room temperature (λ_{exc} = 379 nm).

Table S2. Solution-state photophysical data of **PXZ-Nap** and **PTZ-Nap**.

Solvents	**PXZ-Nap**			**PTZ-Nap**		
	λ_{PL} /nm[a]	τ_{PL} /ns[b]	Φ_{PL}[c] /%	λ_{PL} /nm[a]	τ_{PL} /ns[b]	Φ_{PL}[c] /%
Toluene	493	5.01	7.9	493	4.55	3.5
THF	541	12.39	4.2	555	10.02	2.3
DMF	583	12.49	1.3	597	10.72	1.1

[a]λ_{exc} = 370 nm. [b]λ_{exc} = 379 nm. [c] in air.

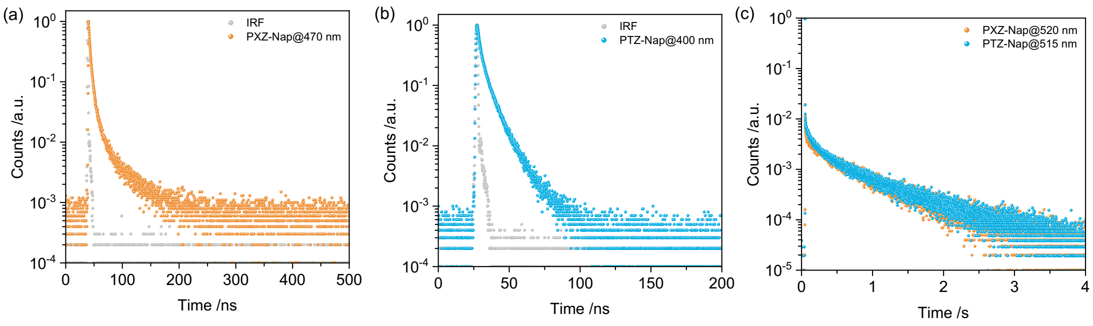

Figure S19. Time-resolved PL decay profiles of (a) 1 wt% **PXZ-Nap** and (b) 1 wt% **PTZ-Nap** in PMMA in air at 298 K. (c) Time-resolved PL decay profiles of 1 wt% **PXZ-Nap** and 1 wt% **PTZ-Nap** in PMMA in vacuum at 77 K (λ_{exc} = 379 nm).

Table S3. Photophysical data of **PXZ-Nap** and **PTZ-Nap** (1 wt% and 10 wt%) in PMMA.

	Fluorescence[a]		RTP[b]		LTP[c]		Φ_{PL} /%		
	λ_{PL} / nm[c]	τ_{PL} /ns[d]	λ_{PL} /nm[c]	τ_{PL} /ms[d]	λ_{PL} /nm[c]	τ_{PL} /ms[e]	air	N_2	Φ_{RTP}[f]
1 wt% **PXZ-Nap**	470	7.55	470	83.3	520	584.2	1.3	1.5	0.2
10 wt% **PXZ-Nap**	495	7.16	475	42.1	525	503.9	2.4	2.9	0.5
1 wt% **PTZ-Nap**	400	5.19	510	66.7	515	628.0	5.3	5.6	0.3
10 wt% **PTZ-Nap**	400<br>505	5.20<br>13.06	500	135.6	515	471.2	3.7	5.8	2.1

[a] Emission in air at 298 K. [b] Emission in vacuum at 298 K. [c] Emission in vacuum at 77 K. [c] λ_{exc} = 370 nm, fluorescence lifetime recorded at emission maximum at 298 K. [d] λ_{exc} = 379 nm, RTP lifetime recorded at emission maximum at 298 K. [e] λ_{exc} = 379 nm, LTP lifetime recorded at emission maximum at 77 K. [f] $\Phi_{RTP} = \Phi_{N2} - \Phi_{air.}$

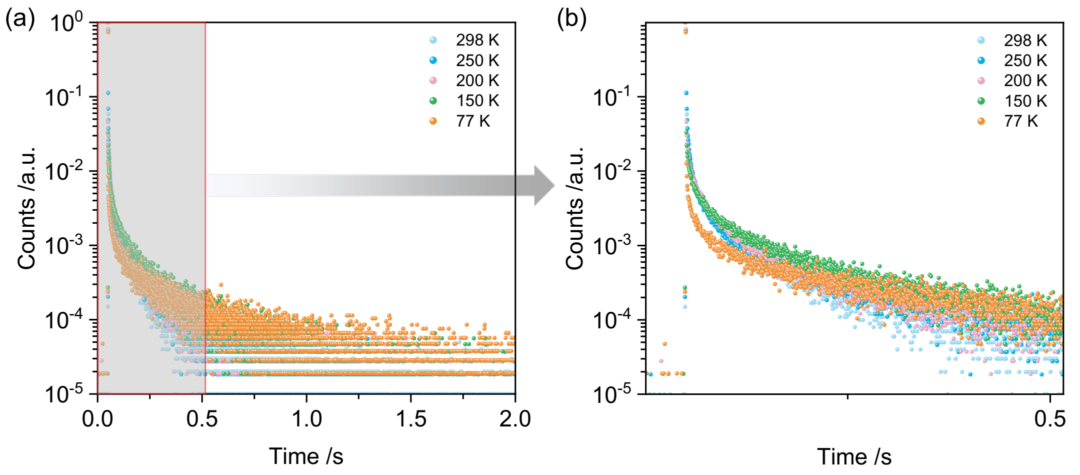

Figure S20. (a) Time-resolved phosphorescence decay profiles and (b) locally zoomed time-resolved phosphorescence decay profiles of 1 wt% **PXZ-Nap** in PMMA at different temperatures at 470 nm (λ_{exc} = 379 nm).

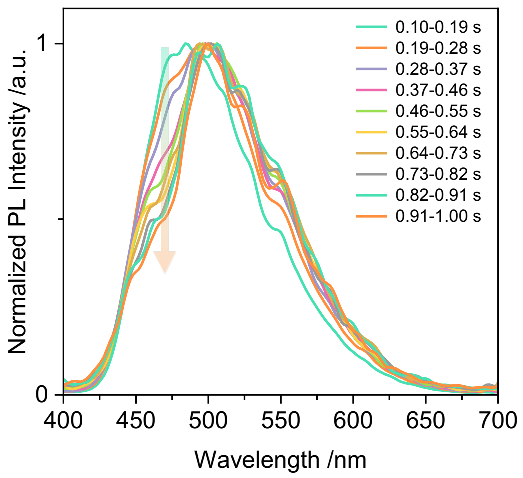

Figure S21. Phosphorescence emission spectra of 1 wt% **PXZ-Nap** in PMMA at 200 K at different time-gated windows (λ_{exc} = 379 nm).

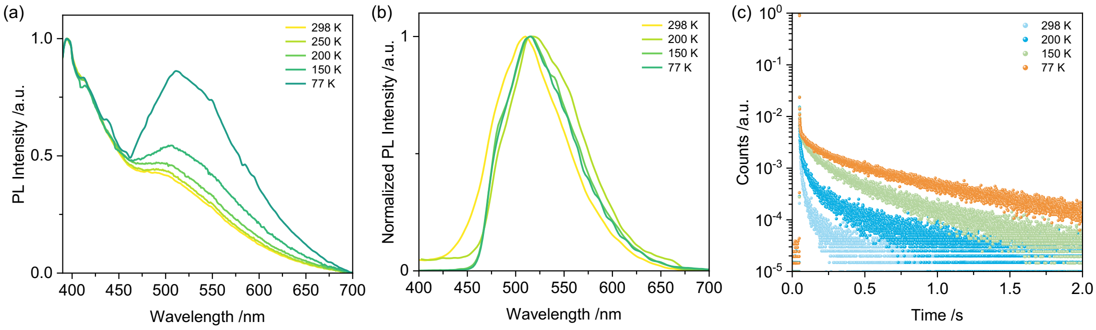

Figure S22. (a) Temperature-dependent steady-state emission spectra of 1 wt% **PTZ-Nap** in PMMA (normalized at 395 nm, λ_{exc} = 370 nm). (b) Temperature-dependent phosphorescence emission spectra of 1 wt% **PTZ-Nap** (time-gated window: 10-200 ms for 298 K; 0.1-1 s for 200 K and 150 K; 0.1-1.5 s for 77 K). (c) Temperature-dependent time-resolved phosphorescence decay profiles of 1 wt% **PTZ-Nap** (λ_{em} = 510 nm, λ_{exc} = 379 nm). The gradually prolonged phosphorescence lifetime can only demonstrate the suppression of nonradiative processes as the temperature decreases. It is difficult to distinguish T_1^H and T_1^L without observing distinct triplet emission bands.

Table S4. Summarized photophysical data of 1 wt% **PXZ-Nap** and 1 wt% **PTZ-Nap** in mCP.

	RTP[a]		LTP[b]		T_1 energy[c]		Φ_{PL}/%	
	λ_{PL} /nm	τ_{PL} /ms	λ_{PL} /nm	τ_{PL} /ms	T_1^H /eV	T_1^L /eV	air	N_2
1 wt% **PXZ-Nap**	490	10.04	512	686.21	2.82	2.56	10.8	26.9
			535	647.21				
1 wt% **PTZ-Nap**	475	20.76	475	1517.84	2.79	2.64	7.7	15.9
	515	24.68						
	550	25.43	512	425.43				

[a] phosphorescence in vacuum at 298 K. [b] phosphorescence in vacuum at 77 K. λ_{exc} = 379 nm. Time-gated windows of RTP and LTP are 10-200 ms and 0.1-2.0 s, respectively. [c] value estimated from the onset of the RTP (T_1^H) and LTP (T_1^L) spectra.

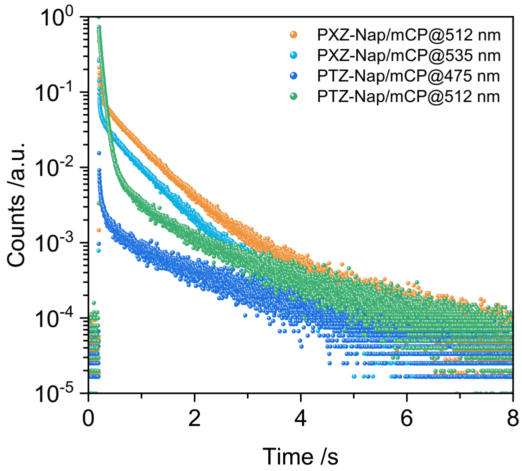

Figure S23. Time-resolved phosphorescence decay profiles of 1 wt% **PXZ-Nap** and 1 wt% **PTZ-Nap** in mCP at 77 K (λ_{exc} = 379 nm).

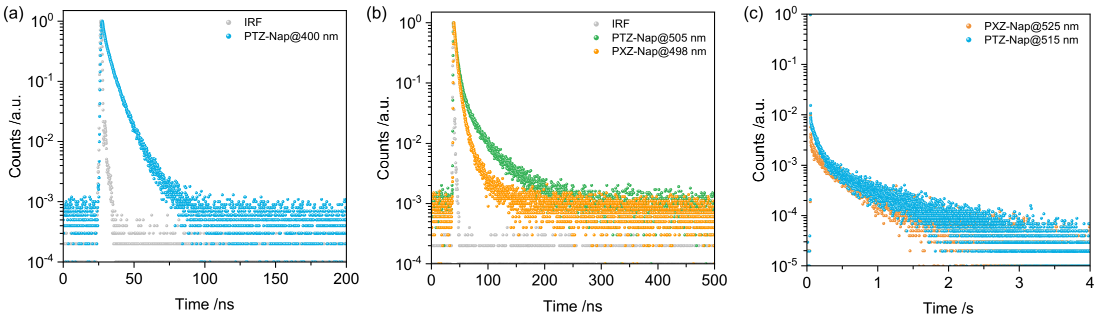

Figure S24. Time-resolved PL decay profiles of (a) 10 wt% **PXZ-Nap** and (b) 10 wt% **PTZ-Nap** in PMMA in air at 298 K. (c) Time-resolved phosphorescence decay profiles of 10 wt% **PXZ-Nap** and 10 wt% **PTZ-Nap** in PMMA in vacuum at 77 K (λ_{exc} = 379 nm).

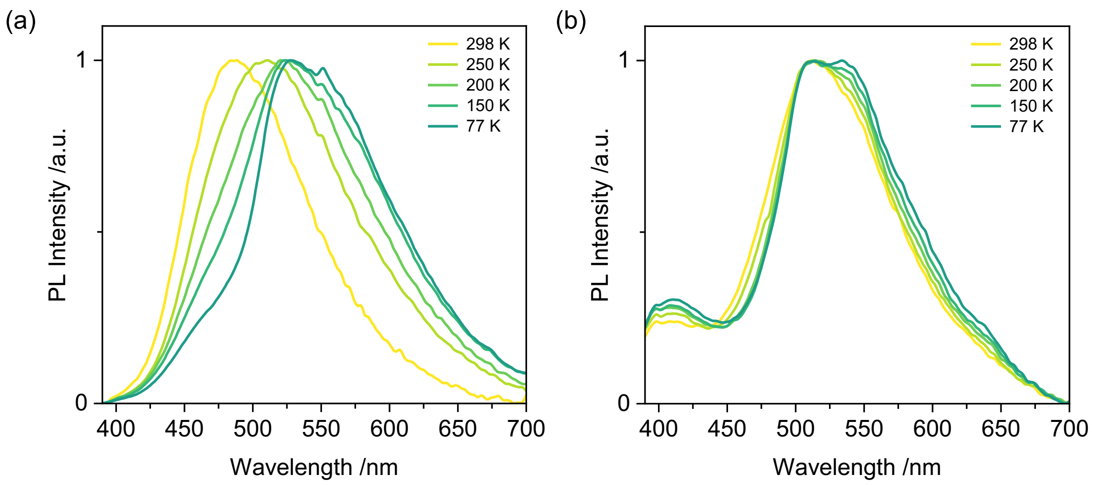

Figure S25. Normalized temperature-dependent steady-state emission spectra of (a) 10 wt% **PXZ-Nap** and (b) 10 wt% **PTZ-Nap** in PMMA (λ_{exc} = 370 nm).

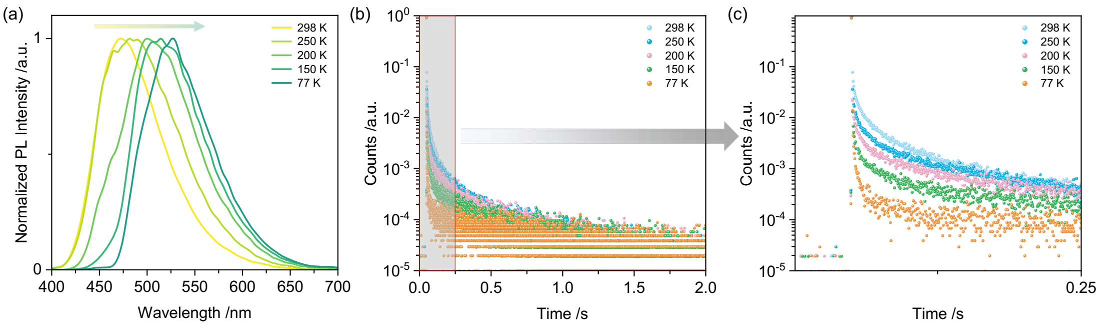

Figure S26. (a) Temperature-dependent phosphorescence emission spectra of 10 wt% **PXZ-Nap** in PMMA. (b) Time-resolved phosphorescence decay profiles and (c) locally zoomed temperature-dependent time-resolved phosphorescence decay profiles of 10 wt% **PXZ-Nap** in PMMA (λ_{em} = 475 nm). The shortened lifetime at 475 nm at 77 K is caused by significantly suppressed T_1^H emission, demonstrated in **Figure S25a**, which is different from TADF.

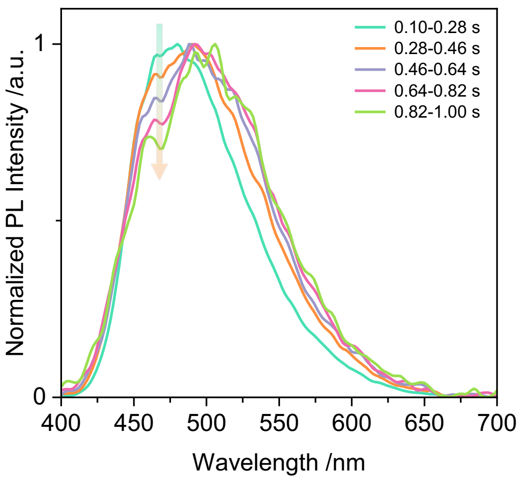

Figure S27. Phosphorescence spectra of 10 wt% **PXZ-Nap** in PMMA at 250 K at various time-gated windows (λ_{exc} = 379 nm).

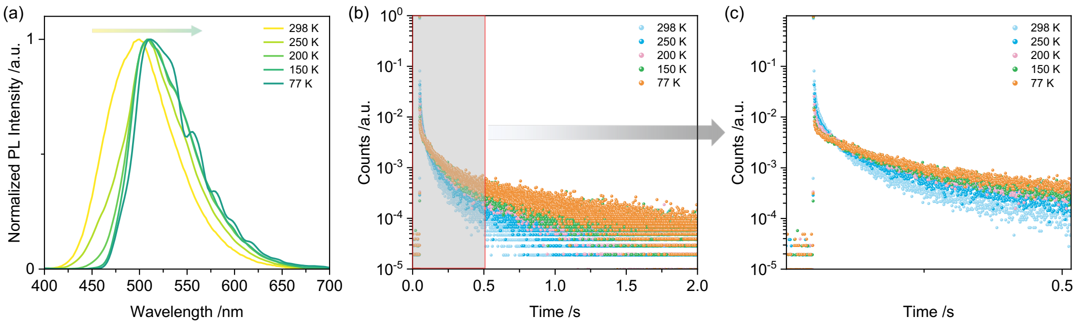

Figure S28. (a) Temperature-dependent phosphorescence spectra of 10 wt% **PTZ-Nap** in PMMA. (b) Times-resolved phosphorescence decay profiles and (c) locally zoomed temperature-dependent time-resolved phosphorescence decay profiles of 10 wt% **PXZ-Nap** in PMMA (λ_{em} = 500 nm, λ_{exc} = 379 nm).

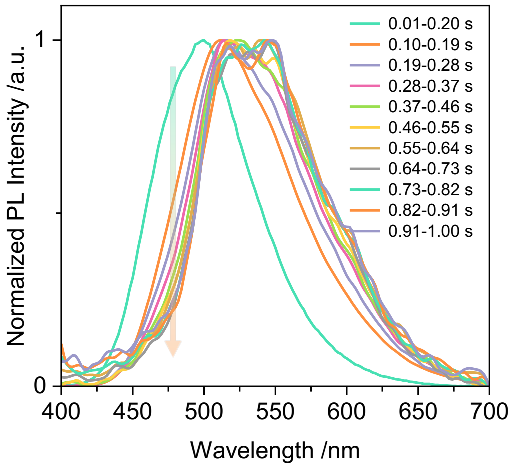

Figure S29. Phosphorescence spectra of 10 wt% **PTZ-Nap** in PMMA at 290 K at various time-gated windows (λ_{exc} = 379 nm).

References

(1) Gomurashvili, Z.; Crivello, J. V. Monomeric and polymeric phenothiazine photosensitizers for photoinitiated cationic polymerization. *Macromolecules* **2002**, *35* (8), 2962-2969.

(2) Connelly, N. G.; Geiger, W. E. Chemical redox agents for organometallic chemistry. *Chem. Rev.* **1996**, *96* (2), 877-910.

(3) Cardona, C. M.; Li, W.; Kaifer, A. E.; Stockdale, D.; Bazan, G. C. Electrochemical considerations for determining absolute frontier orbital energy levels of conjugated polymers for solar cell applications. *Adv. Mater.* **2011**, *23* (20), 2367-2371.

(4) Frisch M. J.; Trucks G. W.; Schlegel H. B.; Scuseria G. E.; Robb M. A.; Cheeseman J. R.; Scalmani G.; Barone V.; Petersson G. A.; Nakatsuji H.; Li X.; Caricato M.; Marenich A. V.; Bloino J.; Janesko B. G.; Gomperts R.; Mennucci B.; Hratchian H. P.; Ortiz J. V.; Izmaylov A. F.; Sonnenberg J. L.; Williams-Young D.; Ding F.; Lipparini F.; Egidi F.; Goings J.; Peng B., Petrone A.; Henderson T.; Ranasinghe D.; Zakrzewski V. G.; Gao J.; Rega N.; Zheng G.; Liang W.; Hada M.; Ehara M.; Toyota K.; Fukuda R.; Hasegawa J.; Ishida M.; Nakajima T.; Honda Y.; Kitao O.; Nakai H.; Vreven T.; Throssell K.; Montgomery J. A.; Jr.; Peralta J. E.; Ogliaro F.; Bearpark M. J.; Heyd J. J.; Brothers E. N.; Kudin K. N.; Staroverov V. N.; Keith T. A.; Kobayashi R.; Normand J.; Raghavachari K.; Rendell A. P.; Burant J. C.; Iyengar S. S.; Tomasi J.; Cossi M.; Millam J. M.; Klene M.; Adamo C.; Cammi R.; Ochterski J. W.; Martin R. L.; Morokuma K.; Farkas O.; Foresman J. B.; Fox D. J. Gaussian, Inc., *Gaussian 16 rev. C.01* Wallingford, CT, 2019.

(5) Adamo, C.; Barone, V. Toward reliable density functional methods without adjustable parameters: The pbe0 model. *J. Chem. Phys.* **1999**, *110* (13), 6158-6170.

(6) Petersson, G.; Tensfeldt, T. G.; Montgomery Jr, J. A complete basis set model chemistry. Iii. The complete basis set-quadratic configuration interaction family of methods. *J. Chem. Phys.* **1991**, *94* (9), 6091-6101.
(7) Dennington, R.; Keith, T. A.; Millam, J. M. Gaussview 6.0. 16. *Semichem Inc.: Shawnee Mission, KS, USA* **2016**.
(8) Gao, X.; Bai, S.; Fazzi, D.; Niehaus, T.; Barbatti, M.; Thiel, W. Evaluation of spin-orbit couplings with linear-response time-dependent density functional methods. *J. Chem. Theory Comput.* **2017**, *13* (2), 515-524.
(9) Crosby, G. A.; Demas, J. N. Measurement of photoluminescence quantum yields. Review. *J. Phys. Chem.* **1971**, *75* (8), 991-1024.
(10) Melhuish, W. H. Quantum efficiencies of fluorescence of organic substances: Effect of solvent and concentration of the fluorescent solute1. *J. Phys. Chem.* **1961**, *65* (2), 229-235.
(11) Hong, J.-S.; Shin, C.-J.; Kim, T.-H.; Kim, K.-S. Triphenylene-based compound as an electroluminescent material and others for organic electro-luminescence device. WO2011081449, 2011.
